# Stability test of canonical correlation analysis for studying brain‐behavior relationships: The effects of subject‐to‐variable ratios and correlation strengths

**DOI:** 10.1002/hbm.25373

**Published:** 2021-02-24

**Authors:** Qingqing Yang, Xinxin Zhang, Yingchao Song, Feng Liu, Wen Qin, Chunshui Yu, Meng Liang

**Affiliations:** ^1^ School of Medical Imaging and Tianjin Key Laboratory of Functional Imaging Tianjin Medical University Tianjin China; ^2^ Department of Radiology and Tianjin Key Laboratory of Functional Imaging Tianjin Medical University General Hospital Tianjin China; ^3^ CAS Center for Excellence in Brain Science and Intelligence Technology Chinese Academy of Sciences Shanghai China

**Keywords:** behaviors, canonical correlation analysis, neuroimaging, reliability, stability

## Abstract

Canonical correlation analysis (CCA), a multivariate approach to identifying correlations between two sets of variables, is becoming increasingly popular in neuroimaging studies on brain‐behavior relationships. However, the CCA stability in neuroimaging applications has not been systematically investigated. Although it is known that the number of subjects should be greater than the number of variables due to the curse of dimensionality, it is unclear at what subject‐to‐variable ratios (SVR) and at what correlation strengths the CCA stability can be maintained. Here, we systematically assessed the CCA stability, in the context of investigating the relationship between the brain structural/functional imaging measures and the behavioral measures, by measuring the similarity of the first‐mode canonical variables across randomly sampled subgroups of subjects from a large set of 936 healthy subjects. Specifically, we tested how the CCA stability changes with SVR under two different brain‐behavior correlation strengths. The same tests were repeated using an independent data set (*n* = 700) for validation. The results confirmed that both SVR and correlation strength affect greatly the CCA stability—the CCA stability cannot be guaranteed if the SVR is not sufficiently high or the brain‐behavior relationship is not sufficiently strong. Based on our quantitative characterization of CCA stability, we provided a practical guideline to help correct interpretation of CCA results and proper applications of CCA in neuroimaging studies on brain‐behavior relationships.

## Graphical abstract

Canonical correlation analysis (CCA) is becoming increasingly popular for studying brain‐behavior relationships. However, the CCA stability in neuroimaging applications has not been systematically investigated. Here, we systematically tested the CCA stability and confirmed that both subject‐to‐variable ratios and correlation strength affect greatly the CCA stability.

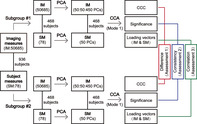

## 1 INTRODUCTION

One of the ultimate goals of neuroimaging studies is to identify relationships between the human brain and the human behavior which can be evaluated by a variety of methods such as Pearson's correlation, multiple regression, and canonical correlation analysis (CCA). Although all these methods aim to examine relationships between different variables, different method fits in different situation. Pearson's correlation analysis is for examining the relationship between two single variables. Multiple regression analysis is for examining relationships between a single variable (i.e., the dependent variable) and a set of variables (i.e., the independent variables). CCA, firstly introduced by Hotelling in 1936 (Hotelling, 1936), is for examining relationships between two sets of variables and can be seen as an extension of correlation analysis and multiple regression analysis (Haigh, Johnson, & Wichern, 1988; Hardoon, Szedmak, & Shawe‐Taylor, 2004; Hotelling, 1936; Kettenring, 1971; Muller, 1982; Skinner & Anderson, 1985). To evaluate the relationship between two sets of variables, CCA seeks the maximal correlation of linear combinations of variables between two sets, and therefore identifies pattern correlations between two sets of variables (Misaki, Wallace, Dankner, Martin, & Bandettini, 2012). CCA is a very appealing method for evaluating the relationships between the human brain and the human behavior because it has several advantages compared with Pearson's correlation analysis and multiple regression analysis. First, both brain imaging measures and behavioral measures are multidimensional in nature, and thus evaluating their relationships naturally requires a multivariate analysis method that can handle multidimensional data on both sides. Indeed, our brain includes many regions that are both correlated and distinct structurally and functionally, and our behavior is also manifested in many correlated but also distinct aspects. Simple one‐to‐one correlation analyses cannot capture their complex relationships on a systematic level. Second, CCA is particularly useful when there are high inter‐correlations between variables of the same set (Lambert, Wildt, & Durand, 1988), which is exactly the case for both brain imaging measures and behavioral measures. Third, similar to the principal component analysis (PCA) (Jolliffe, 1986), CCA decomposes the relationship between two sets of variables into a series of pattern correlations between pairs of canonical variables (i.e., modes of co‐variation) with each mode being a particular linear combination of variables in each set, while ensuring that modes are orthogonal between pairs. Fourth, CCA also allows the identification of variables contributing the most to each mode based on variable loadings, and thus allows a simple and direct interpretation of each mode (Davis, Pierson, & Finch, 2011).

Due to these advantages, CCA is becoming increasingly popular in the identification of relationships between brain imaging measures and behavioral measures in both health (Davis et al., 2011; Perry et al., 2017; Shen et al., 2016; Smith et al., 2015; Tsvetanov et al., 2016; Vidaurre, Smith, & Woolrich, 2017; Wee et al., 2017; Will, Rutledge, Moutoussis, & Dolan, 2017) and diseases (Ball et al., 2017; Cai et al., 2019; Drysdale et al., 2017; Kottaram et al., 2019; Lin et al., 2018; McAnulty et al., 2013; Rodrigue et al., 2018; Viviano et al., 2018). For example, using CCA, Smith et al. identified a strong positive–negative mode of population co‐variation linking brain resting‐state functional connectivity and human behaviors/demographics, showing that subjects were predominantly spread along a single “positive–negative” axis linking lifestyle and psychometric measures to a specific pattern of brain connectivity (Smith et al., 2015). Vidaurre et al. identified two distinct metastates of resting‐state brain network activity—one for sensorimotor and one for higher‐order cognitive functions—and they further used CCA to reveal that the time spent in each metastates related to individual behaviors, particularly cognitive performance and satisfaction (Vidaurre et al., 2017). CCA has also been used to link functional connectivity and clinical symptoms and identified four neurophysiological biotypes of depression (Drysdale et al., 2017). Due to the increasing popularity of CCA in neuroimaging studies, a few review and tutorial papers introducing this method have also been published very recently (Wang et al., 2020; Zhuang, Yang, & Cordes, 2020). All these studies indicate that CCA is an important technique that allows a systematic delineation of brain‐behavior relationships and helps us better understand the neural foundations of human behaviors.

Despite the great potential of CCA in examining brain‐behavior relationships shown in these previous studies, the stability issue in this type of neuroimaging studies has not yet been fully investigated. In univariate analyses such as voxel‐wise comparisons of neuroimaging measures between different experimental conditions or groups in neuroimaging studies (Nature Publishing Group, 2013), concerns about result reliability and reproducibility have been raised increasingly, showing that numerous multiple comparisons and low statistical power often lead to spurious and unreliable results (Button et al., 2013; David et al., 2013; Ioannidis, 2005). Although the multiple comparisons problem is not much of a concern here, the use of multivariate analysis techniques such as CCA may still not be used without restraints. A major concern is the ultra‐high dimensionality of the imaging data due to the commonly known problems of “over‐fitting” and “the curse of dimensionality” (Wang et al., 2020). Indeed, the number of imaging variables (i.e., voxels or connections) is far more than the number of subjects in most scenarios, and consequently, it is often necessary to reduce the dimensionality of the imaging data and sometimes also the behavioral data before performing CCA even though this dimension reduction may result in information loss, so that a high subject‐to‐variable ratio (SVR) can be achieved (Wang et al., 2020; Zhuang et al., 2020). However, given an available number of subjects, how much the data dimensionality should be reduced to obtain a good stability of CCA results is unclear. Furthermore, the CCA stability might be also affected by whether the two sets of variables of interest (i.e., the brain imaging data and the behavioral data) are strongly or weakly correlated with each other. However, it is also unclear how the stability of CCA results changes with the correlation strength between brain imaging data and behavioral data. Addressing these questions is crucial for correct applications of this promising technique in investigations of brain‐behavior relationships.

Therefore, in the present study, we aimed to test the stability of CCA between brain imaging (including structural and functional measures) and subject measures (including demographic and behavioral assessments) data by examining whether similar results could be obtained when using randomly sampled subjects from a homogeneous population for different data dimensionalities and brain‐behavior correlation strengths. More specifically, we used PCA, the most commonly used dimension reduction method in CCA studies (Zhuang et al., 2020), to reduce the brain imaging data to a series of dimensionalities for a fixed number of subjects (i.e., a series of SVRs) before CCA. In addition, we created a “strong correlation” scenario and a “moderate correlation” scenario by including or excluding a few subject measures which showed strong correlations with the brain imaging data. Then, CCA was performed to evaluate the relationship between brain structural (i.e., voxel‐based gray matter volume) or functional (i.e., voxel‐based regional homogeneity) measures and a set of subject measures using different subgroups of subjects (*n* = 468 for each subgroup) sampled from a large data set of healthy young adults (*n* = 936). As the whole data set from which each subgroup is sampled is homogeneous, the CCA results obtained based on each subgroup are expected to be similar if the results are stable. Considering that subject overlapping rate may influence the similarity of the CCA results between different subgroups due to inter‐subject differences, subjects in each subgroup were sampled using a pseudorandom manner to obtain a series of subject overlapping rates between subgroups. Therefore, for each SVR, correlation strength scenario, and subject overlapping rate, the CCA stability was assessed by the similarity of the first CCA mode between randomly sampled subgroups from three aspects: the similarity of canonical correlation coefficients (CCC), the consistency of the statistical significance, and the similarity of canonical variables. Importantly, we further validated our findings by repeating the same analyses in another independent data set (*n* = 700) from the Human Connectome Project (HCP). Note that real data were used to test the CCA stability in the present study as it is very difficult to mimic all aspects of a real data set using simulated data. For example, the variables within each variable set are likely to be related to each other in a very complex manner which is very difficult, if possible at all, to be captured using simulated data. In addition, although a ground‐truth CCC may be predefined and thus available in simulated data, it is difficult to use it to practically guide real data analysis as the true correlation strength between two sets of variables is unknown in real situations.

## 2 MATERIALS AND METHODS

### 2.1 Data sets

#### 2.1.1 Tianjin data set

##### Subjects

A total of 1,104 healthy right‐handed participants (508 males) aged between 18 and 30 years (mean = 23.8, *SD* = 2.4) were included in the Tianjin data set. Further exclusion criteria include MRI contraindications, non‐Han Chinese, left‐handedness or ambidexterity, more than 20 cigarettes smoked by the time of enrollment, history of excessive drinking, drug abuse or dependence, women in pregnancy or menstruation, neuropsychiatric disorders or major physical diseases in present or past, any current medication (include birth control pills), color blindness or color discrimination disorder, brain lesions or structural abnormalities. All participants were recruited from two sites in Tianjin, China, including Tianjin Medical University General Hospital and Tianjin Medical University Cancer Institute and Hospital. From each participant, we collected the structural and functional MRI data along with a series of assessments including basic demographical, environmental, and cognitive questionnaires. Ethical approval was obtained from the medical ethics committee of Tianjin Medical University General Hospital and the medical ethics committee of Tianjin Medical University Cancer Institute and Hospital prior to the study and written informed consent was obtained from each participant before enrollment.

##### Data acquisition

MRI was performed on 3 ‐Tesla GE Discovery MR750 scanners using an eight‐channel head‐receiving coil at Tianjin Medical University General Hospital and Tianjin Medical University Cancer Institute and Hospital. T1‐weighted MRI data were acquired using the following parameters: repetition time (TR) = 8.16 ms, echo time (TE) = 3.18 ms, inversion time (TI) = 450 ms, slice thickness (ST) = 1 mm, flip angle (FA) = 12°, field of view (FOV) = 256 × 256 mm^2^, and voxel size = 1 × 1 × 1 mm^3^. Resting‐state fMRI data were acquired using a gradient‐echo planar imaging (EPI) sequence with TR = 2,000 ms, TE = 30 ms, ST = 3 mm, spacing between slices = 4 mm, FA = 90°, number of slices = 36, and matrix size = 64 × 64. The resting‐state fMRI scan lasted 6.17 mins, resulting in 185 volumes.

Personal information and assessments were also collected for each participant and there are 91 items in total including demographics (e.g., gender, age, education years), cognitive and behavioral performances (e.g., California word learning test II, virtual ball‐tossing game, n‐back working memory task, go‐no go task) and natural and social environmental measures (e.g., childhood trauma questionnaire, urbanization scores). These 91 items are referred to as “subject measures” hereafter, as opposed to “imaging measures.”

##### Image processing

###### Gray matter volume

The structural T1‐weighted images were processed to extract voxel‐wise gray matter volume (GMV) using the Voxel‐Based Morphometry toolbox (VBM8, http://www.neuro.uni-jena.de/vbm8) (Ashburner & Friston, 2000) in the Statistical Parametric Mapping software package (SPM12; Wellcome Department of Imaging Neuroscience, London, UK, http://www.fil.ion.ucl.ac.uk) running on Matlab platform (the MathWorks Inc., Natick, Massachusetts). More specifically, a structural brain template was firstly generated based on the same group of subjects using the high‐dimensional Diffeomorphic Anatomical Registration Through Exponentiated Lie Algebra (DARTEL) algorithm (Ashburner, 2007) implemented in SPM12, and then the structural brain images were spatially normalized to this template and were segmented into the gray matter (GM), white matter (WM) and cerebrospinal fluid (CSF). The normalized gray matter images were modulated for nonlinear transformations to obtain voxel‐wise GMV corrected for global brain volume. The modulated GMV images were then spatially smoothed with a 5 mm full width at half maximum (FWHM) Gaussian kernel. To reduce computational load, the smoothed GMV images were downsampled into a lower resolution of 3 mm × 3 mm × 3 mm^3^ voxel size and only the voxels within a gray matter mask (threshold of gray matter density: 50%) were entered into the subsequent CCA analyses.

###### Resting‐state regional homogeneity

The resting‐state fMRI data of each subject were preprocessed using Data Processing Assistant for Resting‐State fMRI (DPARSF; http://rfmri.org/dparsf) software (Yan & Yufeng, 2010) using the following procedure. The first five volumes were discarded for allowing the stabilization of signal and the adaptation of subjects to the scanning environment. Slice timing and spatial realignment were performed to adjust acquisition time differences across different slices and to adjust head motion between different volumes during scanning, respectively. The images were subsequently coregistered with the high‐resolution T1‐weighted image and then spatially normalized into the Montreal Neurological Institute (MNI) standard space using the unified segmentation‐normalization procedure (the same template as in the VBM analysis was used) and the voxel size of images was resampled to 3 ×  3 × 3  mm^3^. The time course of fMRI signals of each voxel was then denoised using the following steps: linear detrending, regressing out 24 head motion parameters (Friston, Williams, Howard, Frackowiak, & Turner, 1996), WM signal, CSF signal and the global signal, data scrubbing with framewise displacement (FD) ≥ 0.3 mm (spline interpolation) (Power, Barnes, Snyder, Schlaggar, & Petersen, 2012, 2013), and temporal filtering (0.01–0.08 Hz). Finally, regional homogeneity (ReHo) was calculated in a voxel‐wise manner and the resultant ReHo map was standardized (with unit mean) and spatially smoothed with an 8mm FWHM Gaussian kernel for each subject. Similar to GMV images, only the voxels within the gray matter mask were entered into the subsequent CCA analyses.

##### Data quality control

Of 91 subject measures, 4 variables had more than 200 missing values and 9 variables had little variance across subjects as more than 800 subjects had exactly the same value, and thus these 13 subject measures were discarded in the subsequent analyses. Among the remaining 78 subject measures, missing values still existed in 168 subjects and thus these 168 subjects were also discarded. Finally, 78 subject measures of 936 subjects were used in the subsequent CCA analyses. Note that, although data normality is desirable, CCA can accommodate any metric variable without strict dependence on normality (Wang et al., 2020). Therefore, categorical variables (e.g., gender) were also included in the subsequent CCA analysis, as in previous studies (Ball et al., 2017).

#### 2.1.2 HCP data set

##### Subjects

A total of 1,113 healthy participants (aged between 22 and 37 years) with 3‐Tesla T1 anatomical MRI data from the Human Connectome Project (HCP) (Van Essen et al., 2013) were used as an independent data set for results validation.

##### Data acquisition

Data acquisition details can be found in (https://db.humanconnectome.org). In brief, all participants were scanned at Washington University, using a Siemens MR scanner with a 32‐channel receiving head coil. Structural T1‐weighted MRI data were acquired using the following parameters: TR = 2,400 ms, TE = 2.14 ms, TI = 1,000 ms, FA = 8°, FOV = 224 × 224 mm^2^, and voxel size = 0.7 × 0.7 × 0.7 mm^3^. Subject measures, including 494 variables in total, were also collected for each participant, including demographics (e.g., gender, age, education years) and cognitive and behavioral performances (e.g., picture sequence memory task, dimensional change card sort task, Flanker task, oral reading recognition task).

##### Image processing

The image processing procedure for extracting GMV values for the HCP data set was the same as used for Tianjin data set with only one exception that the T1 template used in the spatial normalization was the European template included in VBM8.

##### Data quality control

Of 494 subject measures, 19 variables had more than 200 missing values, 103 variables had little variance across subjects as more than 800 subjects had exactly the same value, 60 variables were the raw options for each item of the Neuroticism/Extroversion/Openness Five‐Factor Inventory (NEO‐FFI), 10 variables were used to assess menstruation in women and missing in all men, 2 variables were bedtime and getting‐up time, 2 variables were zygosity‐related indicators and 8 variables were hormone‐related endocrine indicators, and thus these 204 variables were discarded in the subsequent analyses. Among the remaining 290 subject measures, missing values still existed in 413 subjects and thus these 413 subjects were discarded. Finally, 290 subject measures of 700 subjects were used in the subsequent CCA analyses.

### 2.2 Canonical correlation analysis and its stability assessment using Tianjin data set in a “strong correlation” scenario

CCA is designed to seek the maximal correlation of linear combinations of variables in two sets (Hotelling, 1936). Given the two sets of variables, *X* (e.g., the imaging measures) and *Y* (e.g., the subject measures), CCA seeks two transformation vectors *A1* and *B1* such that the new variables *U1* = *A1* ^*T*^ *X* and *V1 = B1* ^*T*^ *Y* are maximally correlated, where *A1* and *B1* are often called the first pair of canonical vectors or weights and the superscript *T* indicates the operator “transpose”. The new variables *U1* and *V1* are called the first pair of canonical variables. The Pearson correlation coefficient between the canonical variables *U1* and *V1* is called the canonical correlation coefficient (CCC) of the first pair. And this maximal canonical correlation pattern is called the first mode of CCA. Then the second pair of canonical variables *U2* and *V2* that is orthogonal to the first pair can be similarly obtained. And this procedure can continue until up to *p* pairs of canonical variables are identified where *p* is the maximum number of variables between the two sets. Therefore, the first mode represents the pattern with the maximal canonical correlation between two variable sets. In addition, to determine the relationship between each original variable and the canonical variable, the loading of each original variable is often calculated as the correlation coefficient between the original variable and the corresponding canonical variable (i.e., *U1*/*V1*). For each set, the loadings of all variables compose the loading vector. The variables with the highest loadings can be interpreted as most strongly associated with, and thus have the most contributions to, the canonical correlation pattern (i.e., the mode) between the two sets of variables (Ball et al., 2017; Cai et al., 2019; Davis et al., 2011; Smith et al., 2015).

**FIGURE 1:**
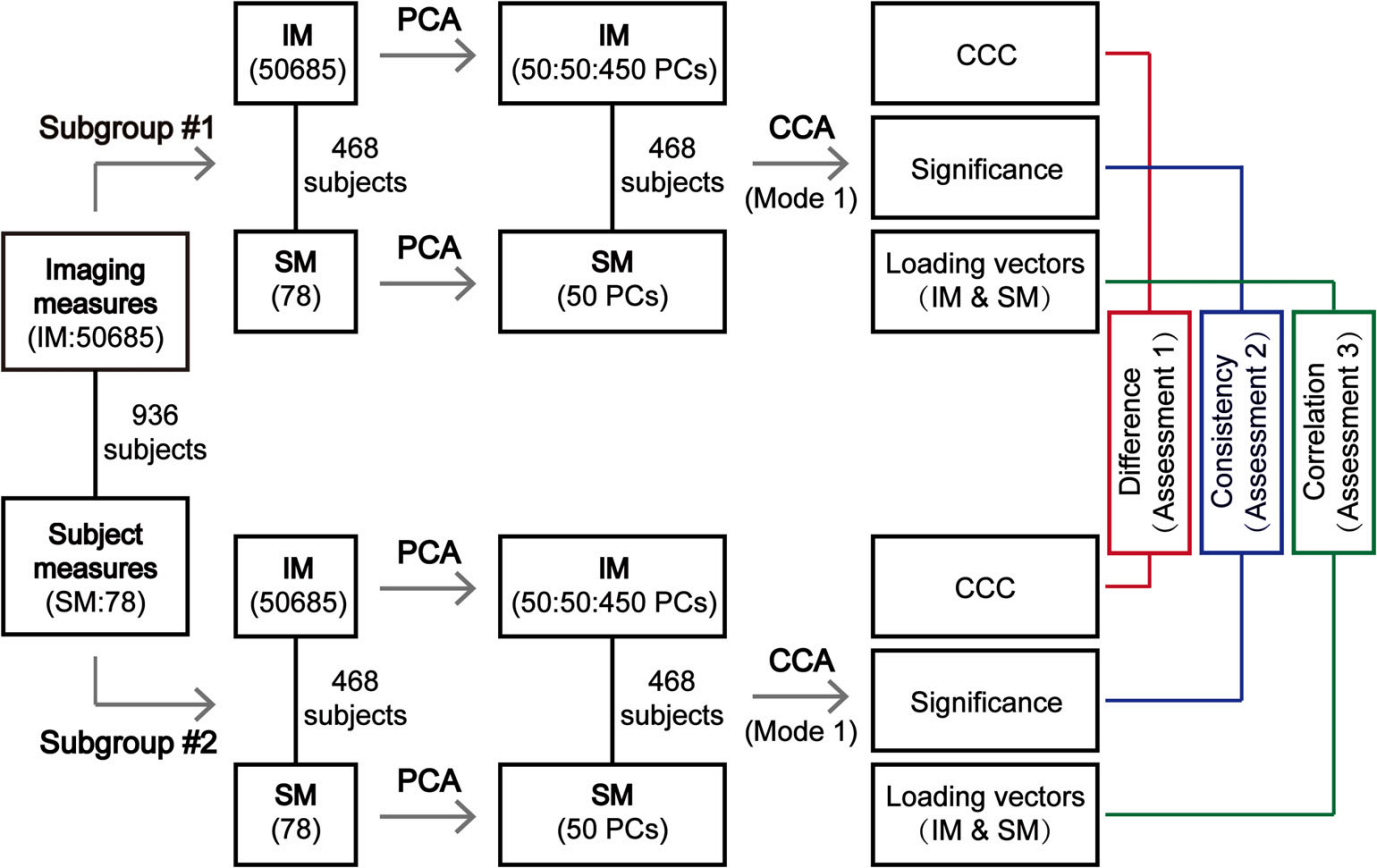
The workflow of CCA implementation in the “main procedure.” The whole Tianjin data set was pseudorandomly split into two subgroups while controlling the subject overlapping rate of the two subgroups ranging from 0 to 450 in increments of 50. To avoid the over‐fitting problem and to test how the number of dimensions affects the CCA results, we reduced the imaging data to a series of dimensionalities ranging from 50 to 450 in increments of 50 using PCA. After PCA, for a given subgroup of subjects, CCA was applied to identify the relationship between brain imaging measures (IM) and subject measures (SM), resulting in the first‐mode CCC, its statistical significance (determined by permutation tests), and two loading vectors (one for brain imaging measures and one for subject measures). Three assessments were performed to test the stability of the CCA results: the differences in CCC (Assessment 1), the consistency of statistical significance (Assessment 2), and the correlation between the loading vectors for each variable set (Assessment 3) between two paired subgroups. This procedure was repeated 1,000 times for each combination of overlapping rate and dimensionality

The procedure of the stability assessments for CCA results is illustrated in the workflow chart in Figure 1 and also as a pseudocode in Figure S1. In detail, to assess the stability of CCA results, before performing CCA, the whole Tianjin data set (*n* = 936) was pseudorandomly split into two subgroups (*n* = 936/2 = 468 for each subgroup, that is, the largest possible sample size of each group allowing zero overlapping subject between the two subgroups) while controlling the subject overlapping rate of the two subgroups. Although there are usually no overlapping subjects in real situations, to test how robust the CCA results would be when the sampled subjects were different, we generated pairs of subgroups with a series of subject overlapping rates ranging from 0 to 450 overlapping subjects with an increment of 50 in each step using the following procedure. To generate two subgroups with 0 overlapping subjects, 468 subjects were randomly selected to form one subgroup and the remaining 468 subjects were used to form the second subgroup; and this procedure was repeated 1,000 times, resulting in 1,000 pairs of subgroups with 0 overlapping subjects. To generate two subgroups with 50 overlapping subjects, 50 subjects were randomly selected and assigned to both subgroups, and 418 subjects (468–50 = 418) were randomly selected from the remaining 886 subjects (936–50 = 886) and assigned to the first subgroup and another 418 subjects were randomly selected from the remaining 468 subjects (936–50−418 = 468) and assigned to the second subgroup; and this procedure was repeated 1,000 times, resulting in 1,000 pairs of subgroups with 50 overlapping subjects. Similarly, we also generated 1,000 pairs of subgroups with 100, 150, up to 450 overlapping subjects. To avoid over‐fitting problem, PCA was applied to imaging data (GMV/ReHo) and subject measure data separately to reduce the data dimensionality. Note that, each variable of the brain imaging data and the subject measure data was standardized to Z score (with zero mean and unit variance) before PCA, and the obtained PCs were standardized to Z scores again before entering CCA. To test how dimensionality affects the CCA stability, we reduced the imaging data to a series of dimensionalities ranging from 50 to 450 with an increment of 50, while the dimensionality of the subject measure data was fixed to 50 since there were only 78 variables in total. This resulted in a series of SVRs from 9.36 (i.e., 468/50) to 1.04 (i.e., 468/450). Here, the SVR was calculated based on the dimensionality of the imaging measures as the dimensionality of the subject measures was kept fixed.

After PCA, for a given subgroup of subjects, CCA was applied to identify the relationship between brain imaging measures and subject measures. Only the first mode, corresponding to the maximal correlation between the two types of variables and thus the most important and the most commonly reported results in a CCA study, was used for the subsequent CCA stability assessments to avoid excessively complicated analyses. The statistical significance of the first‐mode CCC of each subgroup was determined by permutation tests (*n* = 100, in order to reduce the computational load). Here, as the two subgroups of subjects in each pair were randomly selected from a homogeneous data set, the CCA results (i.e., CCC, its statistical significance, and canonical variables) were expected to be similar if they are stable. Therefore, the stability of CCA results was assessed as the similarity between the CCA results obtained from two randomly selected subgroups of subjects in each pair for each subject overlapping rate and each data dimensionality from the following three aspects: (a) the similarity of the first‐mode CCCs obtained from two paired subgroups (Assessment 1): it was measured by the absolute difference of the CCCs obtained from the two subgroups for each pair, and then the mean and the *SD* of the absolute differences in CCC across the 1,000 pairs were calculated; (b) the consistency of the statistical significance of the first‐mode CCCs obtained from two paired subgroups (CCCs were considered to be significant when *p* < .05) (Assessment 2): there are three possible outcomes when comparing the statistical significance of CCCs between the two subgroups for each pair—both were significant, both were insignificant, and one significant but the other insignificant—and the percentage of each outcome was calculated over the 1,000 pairs of subgroups; (c) the similarity of the canonical variables of the first modes (i.e., *U1*/*V1*) obtained from two paired subgroups, assessed for brain imaging measures and subject measures separately (Assessment 3): it was measured by the absolute correlation coefficient of the loading vectors between the two subgroups for each pair and then their mean and the *SD* across the 1,000 pairs were calculated. Note that, the similarity of the first modes of the two subgroups in each pair (i.e., the above third aspect) cannot be directly assessed by calculating the correlation coefficient between the *U1*/*V1* of the first subgroup and the *U1*/*V1* of the second subgroup because the direction of *U1*/*V1* is dependent of the order of the subjects included in a particular subgroup but there is no correspondence between the subjects of the two subgroups. Calculating the correlation coefficient between the loading vectors of the two subgroups avoids this problem because the loading vectors are composed of variables (which are the same for the two subgroups) rather than subjects (which are different for the two subgroups).

### 2.3 Re‐assessment of stability of CCA results using Tianjin data set in a “moderate correlation” scenario

**FIGURE 2:**
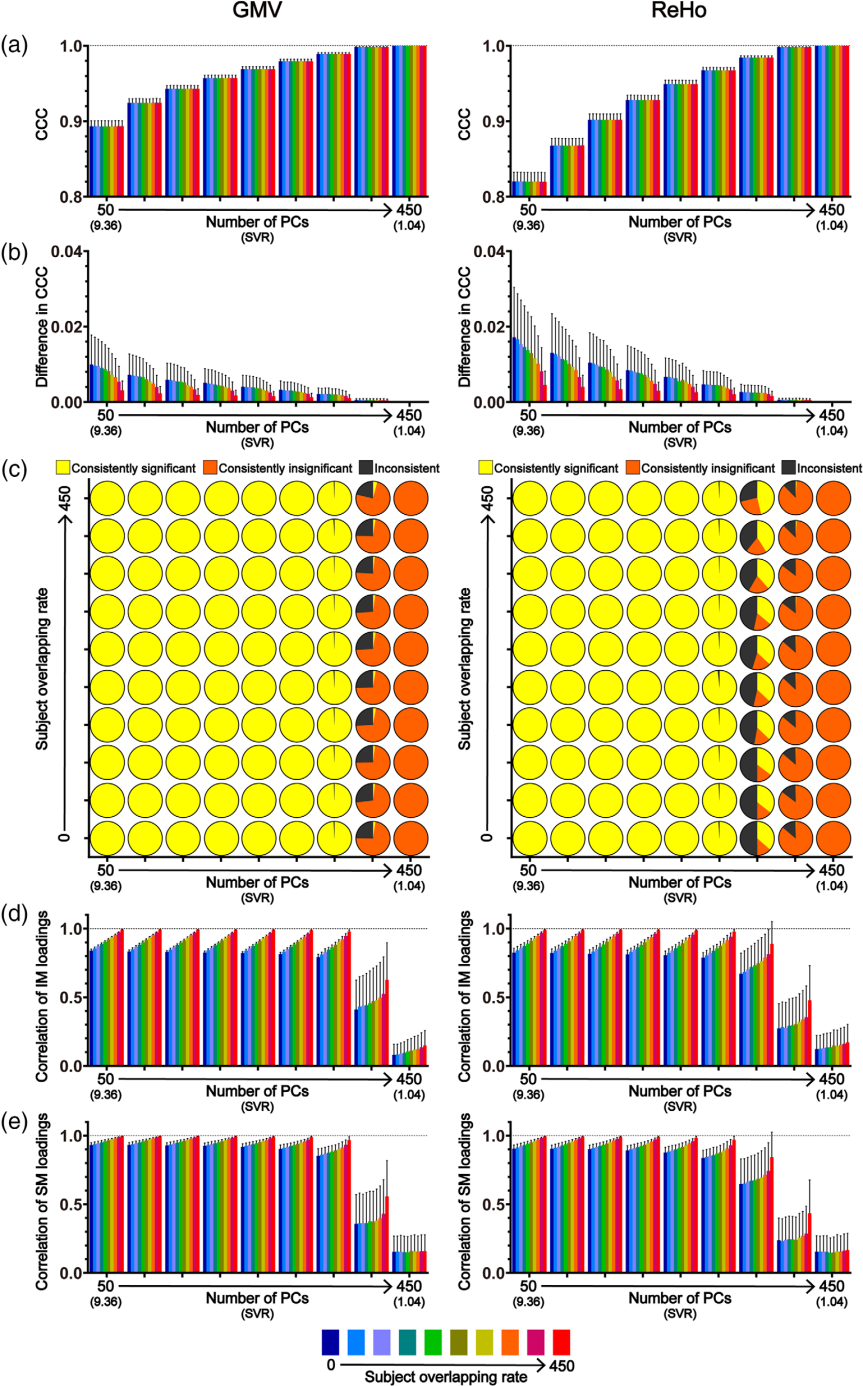
The results of CCA stability assessments using the Tianjin data set in the “main procedure” with all 78 subject measures (i.e., the “strong correlation” scenario). Panel (a) shows the magnitudes of CCCs obtained from 2,000 CCAs for all combinations of subject overlapping rate and data dimensionality. Panel (b) shows the absolute differences in CCCs of 1,000 pairs of CCA. Panel (c) shows the consistency of the statistical significance of CCCs between two subgroups of 1,000 pairs of CCAs. Panels (d) and (e) show the correlation coefficients of the loading vectors between two subgroups of 1,000 pairs of CCAs corresponding to brain imaging measures and those corresponding to subject measures, respectively. The abscissa of all subgraphs represents the dimensionality of imaging measures (i.e., the number of kept PCs, ranging from 50 to 450 with a step of 50) and the corresponding SVR (i.e., the ratio of the sample size to the dimensionality of the imaging measures, ranging from 9.36 to 1.04). The subject overlapping rates between two subgroups of each pair (ranging from 0 to 450 with a step of 50) are color coded. In all bar plots, the height of the bars indicates the mean and the error bars indicate the *SD*. IM, brain imaging measures; SM, subject measures

**FIGURE 3:**
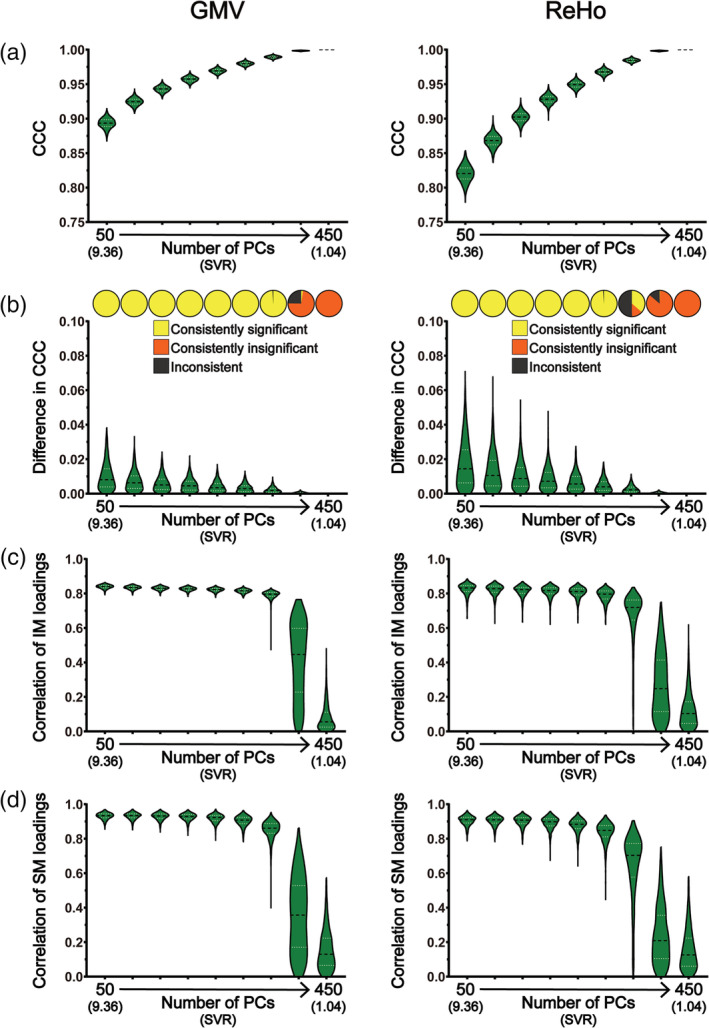
The results of CCA stability assessments using the Tianjin data set in the “main procedure” with all 78 subject measures (i.e., the “strong correlation” scenario) when there are no overlapping subjects between the two subgroups of 1,000 pairs of CCAs. Panel (a) shows the magnitudes of CCCs obtained from 2,000 CCAs for all data dimensionalities. Panel (b) shows the absolute differences in CCCs of 1,000 pairs of CCA (lower part) and the consistency of the statistical significance of CCCs between two subgroups of 1,000 pairs of CCAs (upper part). Panels (c) and (d) show the correlation coefficients of the loading vectors between two subgroups of 1,000 pairs of CCAs corresponding to brain imaging measures and those corresponding to subject measures, respectively. The abscissa of all subgraphs represents the dimensionality of imaging measures (i.e., the number of kept PCs, ranging from 50 to 450 with a step of 50) and the corresponding SVR (i.e., the ratio of the sample size to the dimensionality of the imaging measures, ranging from 9.36 to 1.04). In all smoothed violin plots, the sample median (black dotted line) and quartiles (white dotted line) are superimposed. IM, brain imaging measures; SM, subject measures

As it will be shown in the Results section (Figures 2a and 3a), the CCCs were generally high for all subject overlapping rates (~0.89 for GMV and ~ 0.82 for ReHo when dimensionality = 50 for each variable set, that is, SVR =9.36), indicating a strong relationship between the above used brain imaging measures and subject measures. We noticed that three subject measures (gender, height and weight) had very large loadings (Figures S2B and S3B), indicating that these three subject measures had major contributions to the first‐mode correlation between brain imaging measures and subject measures. To further test whether the correlation strength between the two sets of variables also affects the stability of CCA results and whether the above observations also hold when there is only a moderate correlation between brain imaging measures and subject measures (Lin et al., 2018; Rodrigue et al., 2018), we removed these three subject measures and repeated all the above analyses to re‐assess the stability of CCA results.

### 2.4 Validation using HCP data set

We repeated the same analysis procedure described in Section 2.2 using HCP data set with the following exceptions: as the total number of the subjects was 700 after quality control, each subgroup was composed of 350 subjects, and the subject overlapping rate changed from 0 to 315 with an increment of 35 and the dimensionality changed from 35 to 315 with an increment of 35.

### 2.5 Control procedure: Removing possible contributions of PC inconsistency to CCA stability assessment

Note that, PCA was applied to each subgroup (rather than the whole group of 936 subjects in the Tianjin data set) before CCA in the above procedure (denoted as “main procedure” hereafter). Although the subjects of each subgroup were randomly sampled from a homogeneous population, the obtained PCs for the two subgroups within each pair may not be the same because the subjects composing each subgroup were not the same (Holmes, Huber, & Martin, 2019; Nguyen & Holmes, 2019). Therefore, the inconsistency between the PCs obtained from each subgroup within each pair could be a source of the instability of CCA results. To further test the stability of CCA results without the contribution of the PC inconsistency between subgroups within each pair, we also adopted another procedure (denoted as “control procedure” hereafter)—most analysis steps in the “control procedure” were the same as in the “main procedure” except that, in the control procedure, PCA was first applied to the brain imaging data (GMV/ReHo) and to the subject measures of the whole group of subjects (i.e., 936 subjects), and then the whole group of subjects (in the form of PC scores) was split into two subgroups of subjects; then the subsequent CCA was performed based on the resultant PC scores within each subgroup. The “control procedure” is also illustrated as a pseudocode in Figure S1. In this way, we ensured that the PCs had perfect correspondence across all subgroups and thus we removed the PC inconsistency between subgroups when assessing the stability of CCA results.

## 3 RESULTS

### 3.1 Stability assessment using Tianjin data set in a “strong correlation” scenario

The obtained CCCs are shown in Figure 2a and the results of the CCA stability assessment are shown in Figures 2b–e from three aspects—the similarity of CCCs (Assessment 1; Figure 2b), the consistency of the statistical significance (Assessment 2; Figure 2c) and the correlation between loading vectors (Assessment 3; Figures 2d,e). Figure 2 shows all results for every subject overlapping rates and for every data dimensionality in the “strong correlation” scenario. To show more clearly the variances of the results across the 1,000 pairs of subgroups, we also presented the results when there was no overlapping subjects between two subgroups within each pair (i.e., overlapping rate = 0) separately in Figure 3 in a different form. All results are presented for GMV (the left columns of Figures 2 and 3) and ReHo (the right columns of Figures 2 and 3) separately.

#### 3.1.1 CCC

For each of the 9 data dimensionalities (i.e., the number of PCs, ranging from 50 to 450 in step of 50) and each of the 10 subject overlapping rates (ranging from 0 to 450 in step of 50), 1,000 pairs of subgroups were created and thus CCA between imaging measures (GMV/ReHo) and subject measures was performed 2,000 times (resulting in 2,000 first‐mode CCCs), one for each subgroup. The mean and the *SD* of the 2,000 first‐mode CCCs for all combinations of data dimensionality and subject overlapping rate are shown in Figures 2a and 3a. It shows that, for both GMV (left column) and ReHo (right column), the CCCs were considerably high (all mean CCCs >0.89 for GMV and > 0.82 for ReHo). As expected, the CCC magnitudes gradually increased with the increase of the data dimensionality; in fact, the CCC reached 1 when the data dimensionality reached 400 (i.e., SVR = 1.17) for both GMV and ReHo.

#### 3.1.2 Similarity of CCCs (Assessment 1)

The absolute difference of the two CCCs within each of the 1,000 pairs of subgroups was then calculated, and their mean and *SD* across the 1,000 pairs are shown in Figures 2b and 3b. It shows that the absolute differences in CCCs between two randomly sampled subgroups, along with thier variances, were very small (<0.08) given that all CCCs were high for both GMV (left column) and ReHo (right column). We also found that, in general, the absolute differences in CCCs between two randomly sampled subgroups, along with their variances, gradually decreased with the increase of the subject overlapping rate and with the increase of the data dimensionality.

#### 3.1.3 Consistency of the statistical significance of CCCs (Assessment 2)

The consistency of the statistical significance of CCCs was represented by the percentages of the three possible outcomes (both were significant, both were insignificant, and one significant but the other insignificant) over the 1,000 pairs of subgroups shown in the pie charts in Figure 2c and in the upper part of Figure 3b. Note that, the most stable results would be consistently significant for all 1,000 pairs or consistently insignificant for all 1,000 pairs. We found that, for GMV (Figure 2c, left column), for all subject overlapping rates (i.e., all rows), the CCCs were consistently significant for data dimensionalities ranging from 50 to 300 and were consistently insignificant when data dimensionality increased to 450 but the inconsistency of the statistical significance started to appear when the data dimensionality increased to 350 and became obvious when the dimensionality increased to 400. Similar results were observed for ReHo except that the inconsistency of the statistical significance started to appear when the data dimensionality increased to 300 and became obvious when the dimensionality increased to 350 and 400 (almost half of the 1,000 pairs had inconsistent significance for the dimensionality of 350).

#### 3.1.4 Correlation between loading vectors (Assessment 3)

The similarity of the canonical variables of the first modes (i.e., *U1*/*V1*) obtained from the two subgroups within each of the 1,000 pairs was measured by the absolute correlation coefficient of the loading vectors between two subgroups for brain imaging measures and subject measures separately. Their mean and *SD* across the 1,000 pairs for all combinations of data dimensionality and subject overlapping rate are shown in Figures 2d and 3c for brain imaging measures and in Figures 2e and 3d for subject measures. It shows that, for both brain imaging measures and subject measures and for both GMV (left column) and ReHo (right column), the correlation coefficients between loading vectors for all subject overlapping rates were generally high (> 0.6) when the data dimensionality was less than 400 but decreased dramatically, along with increased variance, when the dimensionality further increased (i.e., ≥ 400). Especially from Figures 3c,d, we can see that the range of the correlation coefficients was extremely wide, varying from 0 to nearly 0.8 when the data dimensionality equals 400, for both brain imaging measures and subject measures. We also found that the values of the correlation coefficients between loading vectors increased with the increase of the subject overlapping rate for all data dimensionalities, for both brain imaging measures (Figure 2d) and subject measures (Figure 2e) and for both GMV (left column) and ReHo (right column). To examine how the loadings themselves changed with the change of data dimensionality, we also presented the loadings of each variable and the absolute differences in loadings of each variable between two paired subgroups across the 1,000 pairs (presented in the form of mean and *SD*) when the subject overlapping rate was 0 in Figures S2A and S3A (for brain imaging measures) and Figures S2B and S3B (for subject measures). We found that, for both brain imaging measures and subject measures, the loadings and the absolute differences in loadings between paired subgroups were fairly stable when the data dimensionality was less than 400 for GMV or less than 350 for ReHo; however, the absolute values of loadings dramatically decreased and the variance of loadings and the absolute differences in loadings dramatically increased when the dimensionality reached 400 for GMV or 350 for ReHo. To further examine whether the same voxels (i.e., brain areas) were detected to have robust loadings in both subgroups of each pair, we also tested the consistency of the significance of the voxel loadings between the two subgroups of each pair. The significance of the voxel loadings was determined using bootstrap testing and the detailed procedure is provided in the Supplemental Methods (Additional Analysis 1). This additional analysis was performed using the GMV data of the Tianjin data set for each SVR. We found that, only for low data dimensionalities (i.e., < 150) in the “strong correlation” scenario, some voxels showed consistently significant loadings in both subgroups and these voxels were mainly located in the bilateral thalamus, bilateral caudate and cerebellum (Figure S4).

### 3.2 Re‐assessment of CCA stability using Tianjin data set in a “moderate correlation” scenario

**FIGURE 4:**
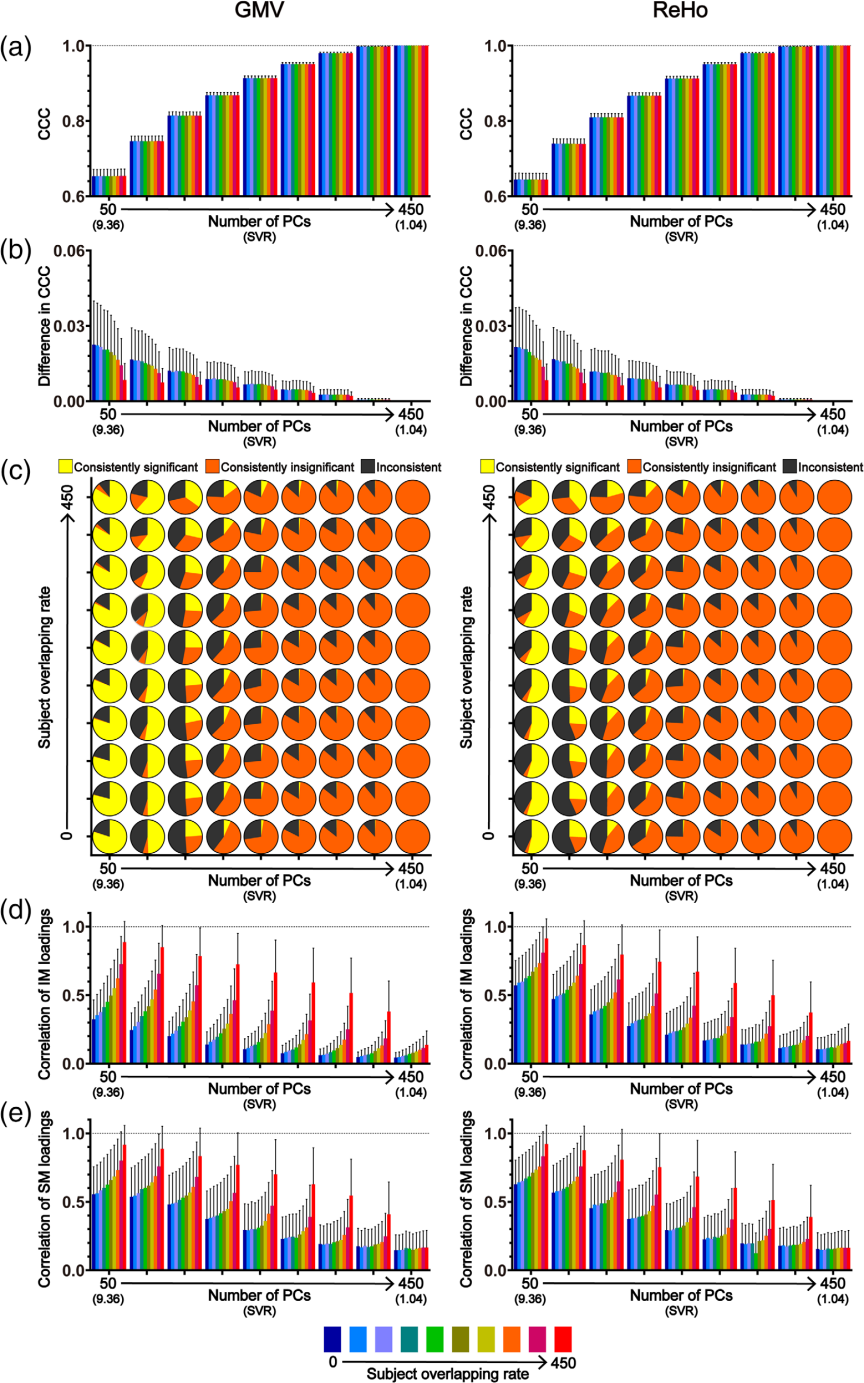
The results of CCA stability assessments using the Tianjin data set in the “main procedure” with 75 subject measures (i.e., the “moderate correlation” scenario). Panel (a) shows the magnitudes of CCCs obtained from 2,000 CCAs for all combinations of subject overlapping rate and data dimensionality. Panel (b) shows the absolute differences in CCCs of 1,000 pairs of CCA. Panel (c) shows the consistency of the statistical significance of CCCs between two subgroups of 1,000 pairs of CCAs. Panels (d) and (e) show the correlation coefficients of the loading vectors between two subgroups of 1,000 pairs of CCAs corresponding to brain imaging measures and those corresponding to subject measures, respectively. The abscissa of all subgraphs represents the dimensionality of imaging measures (i.e., the number of kept PCs, ranging from 50 to 450 with a step of 50) and the corresponding SVR (i.e., the ratio of the sample size to the dimensionality of the imaging measures, ranging from 9.36 to 1.04). The subject overlapping rates between two subgroups of each pair (ranging from 0 to 450 with a step of 50) are color coded. In all bar plots, the height of the bars indicates the mean and the error bars indicate the *SD*. IM, brain imaging measures; SM, subject measures

**FIGURE 5:**
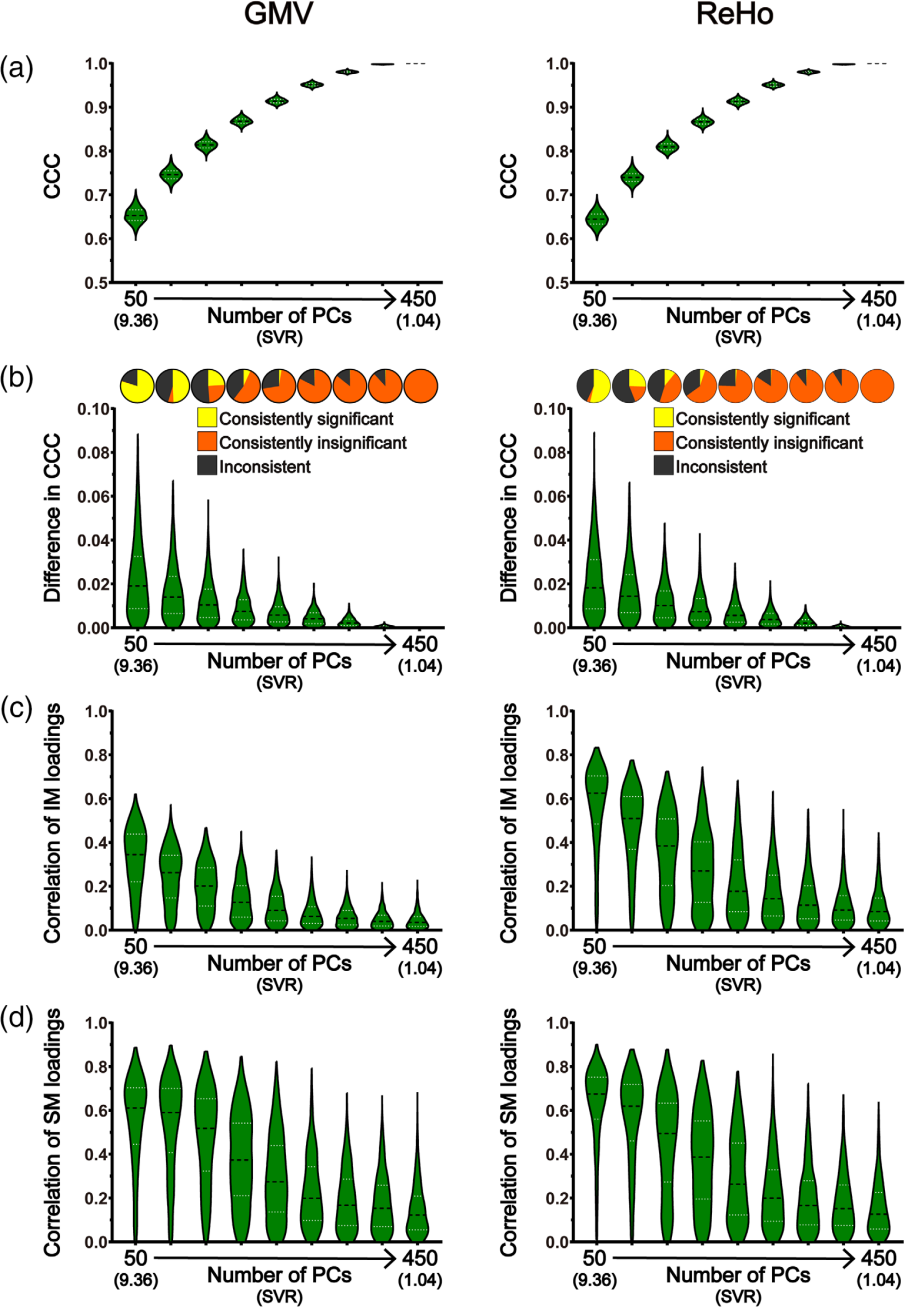
The results of CCA stability assessments using the Tianjin data set in the “main procedure” with 75 subject measures (i.e., the “moderate correlation” scenario) when there are no overlapping subjects between the two subgroups of 1,000 pairs of CCAs. Panel (a) shows the magnitudes of CCCs obtained from 2,000 CCAs for all data dimensionalities. Panel (b) shows the absolute differences in CCCs of 1,000 pairs of CCA (lower part) and the consistency of the statistical significance of CCCs between two subgroups of 1,000 pairs of CCAs (upper part). Panels (c) and (d) show the correlation coefficients of the loading vectors between two subgroups of 1,000 pairs of CCAs corresponding to brain imaging measures and those corresponding to subject measures, respectively. The abscissa of all subgraphs represents the dimensionality of imaging measures (i.e., the number of kept PCs, ranging from 50 to 450 with a step of 50) and the corresponding SVR (i.e., the ratio of the sample size to the dimensionality of the imaging measures, ranging from 9.36 to 1.04). In all smoothed violin plots, the sample median (black dotted line) and quartiles (white dotted line) are superimposed. IM, brain imaging measures; SM, subject measures

In the above analyses which showed a strong correlation between brain imaging measures and subject measures (~0.89 for GMV and ~0.82 for ReHo when dimensionality = 50 for each variable set), we noticed that three subject measures (gender, height and weight) had very large loadings (absolute values > 0.75; Figures S2B and S3B) and all other subject measures had much smaller loadings (absolute values < 0.2), indicating that these three subject measures had major contributions to the first‐mode correlation between brain imaging measures and subject measures. Figures 4, 5, S5, and S6 show the results of stability assessment after removing gender, height and weight from the subject measures (analogue to Figures 2, 3, S2, and S3). After removing these three subject measures, we observed a clear decrease of CCCs especially for the dimensionalities lower than 200—for example, the CCCs decreased from ~0.89 to ~0.65 for GMV and from ~0.82 to ~0.64 for ReHo when there was no overlap between paired subgroups for the dimensionality of 50 (Figures 4a and 5a). Similar to before, we also found that the values of CCCs increased with the increase of the data dimensionality and reached 1 when the data dimensionality reached 400 for both GMV and ReHo.

We found that, after removing the three subject measures, although many trends of how these stability measures change with the subject overlapping rate and with the data dimensionality remained the same, several differences between the two sets of results (Figures 4, 5, S5, and S6 vs. Figures 2, 3, S2, and S3) are also clearly noticeable. First, the absolute differences of CCCs between paired subgroups and their corresponding variances (across the 1,000 pairs) were generally larger in the “moderate correlation” scenario (Figures 4b and 5b) than those in the “strong correlation” scenario (Figures 2b and 3b)—taking the GMV results under the condition of the overlapping rate of 0 with a dimensionality of 50 as an example, the mean of the absolute differences was ~0.008 and ranged from 0 to 0.04 in the “strong correlation” scenario (Figure 3b) whereas the mean of the absolute differences was about 25 times larger (~0.02, ranging from 0 to 0.09) in the “moderate correlation” scenario (Figure 5b). Second, in contrast with the results in the “strong correlation” scenario showing that CCCs were consistently significant for lower dimensionalities ranging from 50 to 300 and their significance only started to become inconsistent when the dimensionality increased to 350 (i.e., SVR = 1.33), CCC's significance was inconsistent even for the lowest dimensionality (i.e., dimensionality = 50, SVR = 9.36) for both GMV and ReHo in the “moderate correlation” scenario (Figure 4c). To test whether the observed inconsistency of CCC significance was caused by nonoptimal estimation of null distributions due to a relatively small number of permutations (*n* = 100), we repeated the same analyses for GMV using 10,000 permutations. We observed similar results, shown in Figure S7, indicating that inconsistent significance of CCCs could still exist for a very large number of permutations. Third, in contrast with the results in the “strong correlation” scenario showing that the correlations between loading vectors were generally high when the dimensionality was less than 400 (i.e., SVR = 1.17), the correlation between loading vectors became much weaker with a much larger variance in the “moderate correlation” scenario (Figures 4d,e and 5c,d)—taking the loadings of GMV under the condition of the overlapping rate of 0 with a dimensionality of 50 as an example, the mean of the correlation coefficients was ~0.84 and ranged from 0.79 to 0.86 in the “strong correlation” scenario (Figure 3c) whereas the mean of the correlation coefficients was ~0.32 and ranged from 0 to 0.62 in the “moderate correlation” scenario (Figure 5c). Fourth, for both brain imaging measures and subject measures, the loadings were less stable (i.e., larger mean and variance of the differences in loadings between paired subgroups across the 1,000 pairs) especially for the variables with relatively large loadings for all data dimensionalities in the “moderate correlation” scenario (Figures S5 and S6) compared with those in the “strong correlation” scenario (Figures S2 and S3). Furthermore, in Additional Analysis 1 for the "moderate correlation" scenario, no voxels showed consistently significant loadings between paired subgroups, which is also in contrast with the "strong correlation" scenario. To test whether more stable results will be obtained if the dimensionality of the imaging measures was further reduced, four extra dimensionalities (10, 20, 30, and 40) of the imaging data were tested and the corresponding results are shown in Figures S8‐S10. We found that the CCA results were still instable even for these extremely low dimensionalities.

### 3.3 Validation using HCP data set

**FIGURE 6:**
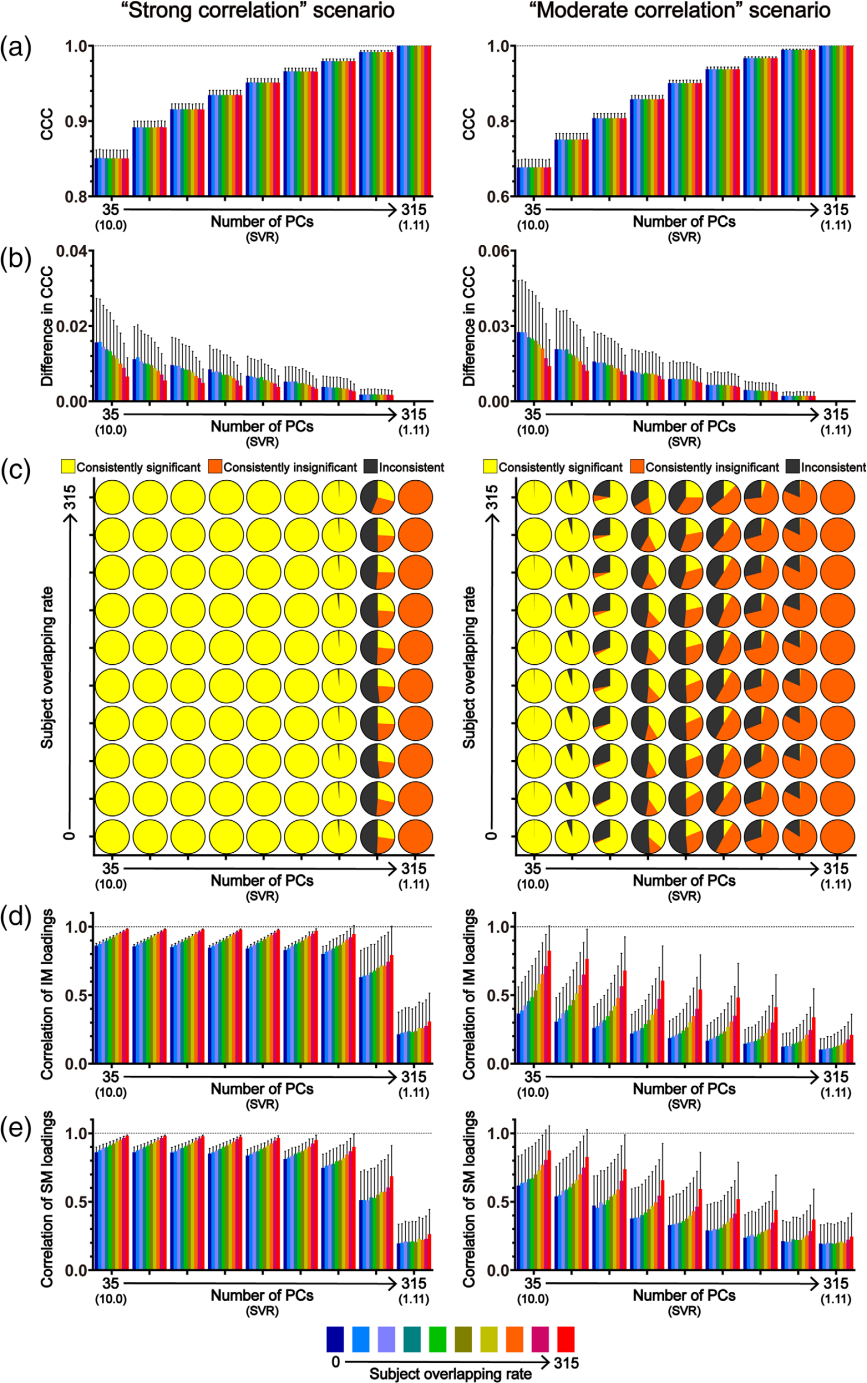
The results of CCA stability assessments using the HCP data set in the “main procedure” with 290 subject measures (i.e., the “strong correlation” scenario; the left column) and with 246 subject measures (i.e., the “moderate correlation” scenario; the right column). Panel (a) shows the magnitudes of CCCs obtained from 2,000 CCAs for all combinations of subject overlapping rate and data dimensionality. Panel (b) shows the absolute differences in CCCs of 1,000 pairs of CCA. Panel (c) shows the consistency of the statistical significance of CCCs between two subgroups of 1,000 pairs of CCAs. Panels (d) and (e) show the correlation coefficients of the loading vectors between two subgroups of 1,000 pairs of CCAs corresponding to brain imaging measures and those corresponding to subject measures, respectively. The abscissa of all subgraphs represents the dimensionality of imaging measures (i.e., the number of kept PCs, ranging from 35 to 315 with a step of 35) and the corresponding SVR (i.e., the ratio of the sample size to the dimensionality of the imaging measures, ranging from 10.0 to 1.11). The subject overlapping rates between two subgroups of each pair (ranging from 0 to 315 with a step of 35) are color coded. In all bar plots, the height of the bars indicates the mean and the error bars indicate the *SD*. IM, brain imaging measures; SM, subject measures

**FIGURE 7:**
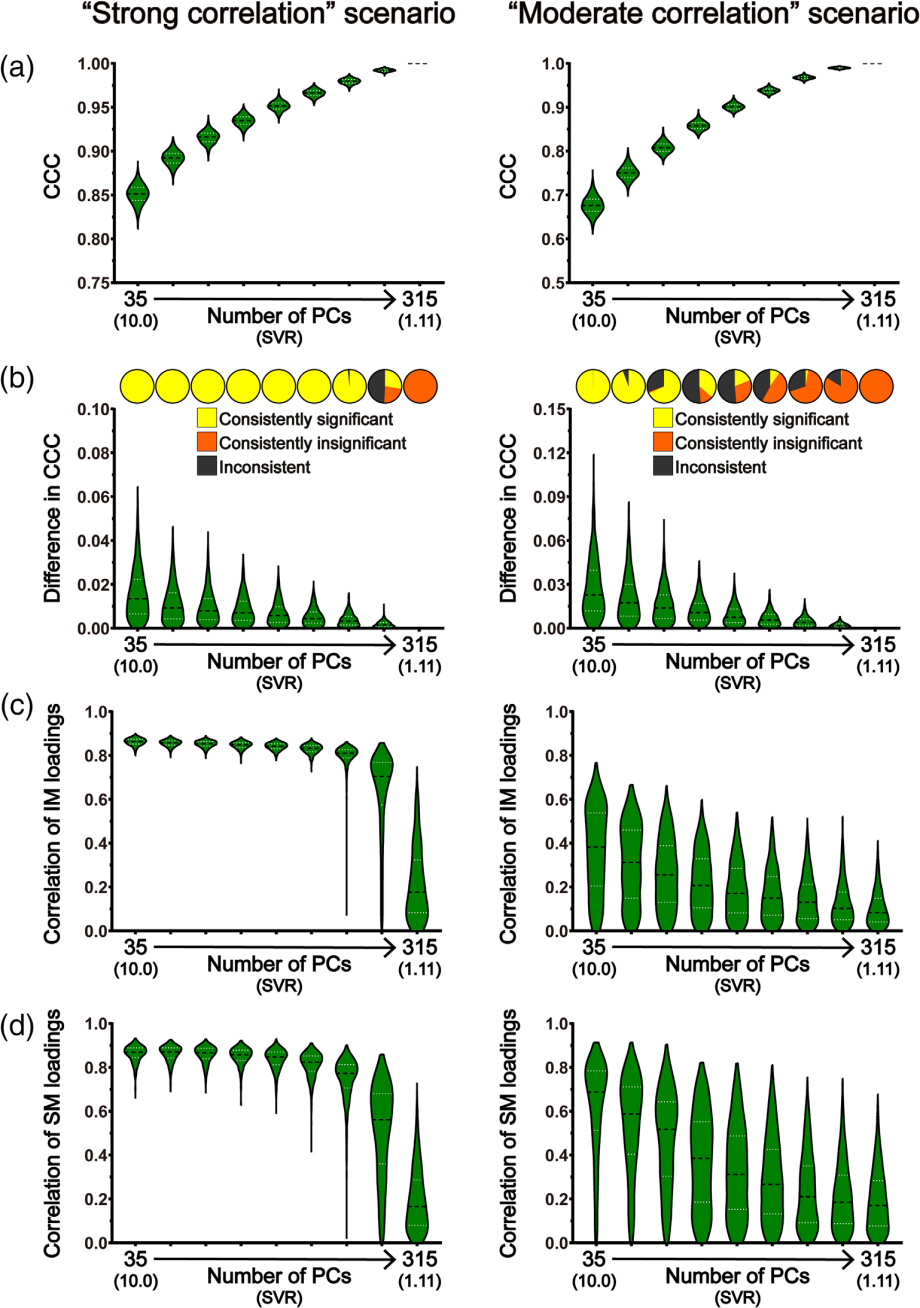
The results of CCA stability assessments using the HCP data set in the “main procedure” with 290 subject measures (i.e., the “strong correlation” scenario; the left column) and with 246 subject measures (i.e., the “moderate correlation” scenario; the right column) when there are no overlapping subjects between the two subgroups of 1,000 pairs of CCAs. Panel (a) shows the magnitudes of CCCs obtained from 2,000 CCAs for all data dimensionality. Panel (b) shows the absolute differences in CCCs of 1,000 pairs of CCAs (lower part) and the consistency of the statistical significance of CCCs between two subgroups of 1,000 pairs of CCAs (upper part). Panels (c) and (d) show the correlation coefficients of the loading vectors between two subgroups of 1,000 pairs of CCAs corresponding to brain imaging measures and those corresponding to subject measures, respectively. The abscissa of all subgraphs represents the dimensionality of imaging measures (i.e., the number of kept PCs, ranging from 35 to 315 with a step of 35) and the corresponding SVR (i.e., the ratio of the sample size to the dimensionality of the imaging measures, ranging from 10.0 to 1.11). In all smoothed violin plots, the sample median (black dotted line) and quartiles (white dotted line) are superimposed. IM, brain imaging measures; SM, subject measures

All the above analyses were repeated using the HCP data set in a “strong correlation” scenario as well as a “moderate correlation” scenario (only GMV was used because very similar stability assessment results were observed for GMV and ReHo when using Tianjin data set). When using all subject measures, we observed very similar results with those obtained using Tianjin data set in the “strong correlation” scenario (Figures 6 and 7, left column; Figure S11): (a) there was a strong correlation between brain imaging measures and subject measures (all mean CCCs > 0.85 for GMV) and the CCC magnitudes gradually increased with the increase of the data dimensionality (Figures 6a and 7a, left column); (b) the absolute differences in CCCs between two randomly sampled subgroups, along with their variances, were very small (Figures 6b and 7b, left column); (c) for all subject overlapping rates, the CCCs were consistently significant for data dimensionalities ranging from 35 to 210 and were consistently insignificant when data dimensionality increased to 315 but the inconsistency of the statistical significance appeared when the data dimensionality increased to 245 (Figure 6c, left column); (d) for both brain imaging measures and subject measures, the correlation coefficients between loading vectors for all subject overlapping rates were generally high (> 0.6) when the data dimensionality was less than 245 but clearly decreased, along with increased variance, when the dimensionality further increased (i.e., ≥ 245) (Figures 6d,e and 7c,d, left column). Then, we also created a “moderate correlation” scenario for the HCP data set by only retaining 246 subject measures with loadings less than 0.2 in the “strong correlation” scenario, as did with Tianjin data set. We found that the CCCs decreased clearly (~0.68 for the overlapping rate of 0 and dimensionality of 35; Figures 6a and 7a, right column), and the stability of the CCA results was also dropped clearly (Figures 6b–e and 7b–d, right column; Figure S12): (a) the absolute differences of CCCs between paired subgroups and their corresponding variances were generally larger (Figures 6b and 7b, right column) than those in the “strong correlation” scenario (Figures 6b and 7b, left column); (b) CCC's significance was inconsistent even for the lowest dimensionality (i.e., dimensionality = 35, SVR = 10) (Figures 6c and 7b, right column); (c) the correlations between loading vectors became much weaker with a much larger variance (Figures 6d‐e and 7c‐d, right column).

### 3.4 Stability assessment using the control procedure

When controlling for the possible contribution of PC inconsistency to the CCA stability assessment in the “control procedure” using Tianjin data set, we found almost identical results in the “strong correlation” scenario (Figures S13‐S16, as compared with Figures 2, 3, S2, and S3). Removing the three subject measures with the highest loadings (i.e., gender, height and weight) resulted in a moderate correlation (Figures S17A and S18A), and the results of the CCA stability assessments (Figures S17‐S20) were very similar (i.e., generally instable) with those obtained in the “moderate correlation” scenario in the “main procedure,” although a few differences were also noticeable. For example, for GMV, the consistency rate of the statistical significance of CCCs and the correlation between loading vectors increased in the “control procedure” for the dimensionality of 50. For ReHo, the consistency rate of the statistical significance of CCCs and the correlation between loading vectors decreased in the “control procedure” for the dimensionality of 50. To test whether more stable results could be obtained if the dimensionality was further reduced in the “control procedure,” four extra dimensionalities (10, 20, 30, and 40) of the imaging data were also tested and the corresponding results are shown in Figures S21‐S23. We found that the CCA results were still instable even for these extremely low dimensionalities, which was very similar to what we observed in the “main procedure.”

## 4 DISCUSSION

Despite the growing popularity of CCA in neuroimaging studies to investigate the relationship between brain and behavior in humans, the stability of the results in such applications has not yet been fully characterized. In the present study, we examined how similar the CCA results were between subgroups of subjects randomly sampled from a large homogeneous data set (*n* = 936) when changing data dimensionality, correlation strength between brain imaging measures and subject measures, and subject overlapping rate. The stability of the CCA results was assessed systematically from three aspects: the similarity of the first‐mode CCC (Assessment 1), the consistency of the corresponding statistical significance (Assessment 2) and the similarity of the first‐mode canonical variables (Assessment 3) between paired subgroups. Importantly, we repeated all analyses using two different brain imaging measures (i.e., a structural measure GMV and a functional measure ReHo) and also in a second independent data set (*n* = 700) to validate the results. We observed similar results using structural (i.e., GMV) and functional (i.e., ReHo) imaging measures in two independent data sets, with and without the possible confound of PC inconsistency. The main findings are summarized as follows. First, both the data dimensionality (i.e., the SVR) and the correlation strength between brain imaging measures and subject measures affect the CCA stability considerably—the stability of CCA results decreased with the increase of data dimensionality and with the decrease of the correlation strength between the two sets of measures. Specifically, when the correlation strength was strong (Tianjin data set: ~0.89 for GMV and ~ 0.82 for ReHo at a SVR of 9.36; HCP data set: ~0.85 for GMV at a SVR of 10), the CCA results were generally stable as long as the data dimensionality was considerably below the sample size (Tianjin data set: SVR > 468/350 = 1.34 for GMV and SVR > 468/300 = 1.56 for ReHo; HCP data set: SVR > 350/245 = 1.43); however, the stability of CCA results cannot be guaranteed if the correlation between the two sets of variables was only moderate (Tianjin data set: ~0.65 for GMV and ~0.64 for ReHo at a SVR of 9.36; HCP data set: ~0.68 for GMV at a SVR of 10) even for a very high SVR (> 10: 468/40 = 11.7, 468/30 = 15.6, 468/20 = 23.4, 468/10 = 46.8)—in our case, for a sample size of 468 of Tianjin Data set, the CCA results were still instable when the dimensionality of imaging measures was no higher than 50 with the dimensionality of subject measures being fixed to 50. In other words, the CCA results are expected to be stable only when the SVR is high and, at the same time, the correlation between the investigated brain imaging measures and subject measures is strong. Second, if the above stability requirements (i.e., high SVR and strong correlation strength) were not met, even just a slight change of subjects in the data to be analyzed could change the CCA results greatly; that was, for an insufficient SVR or a moderate correlation, the CCA results could still be instable even if the subjects in the two subgroups were highly overlapped (e.g., Tianjin data set: 450 of 468 subjects were identical, see the upper row of Figure 4c; HCP data set: 315 out of 350 subjects were identical, see the upper row of the right column in Figure 6c). Third, the CCC, as expected, gradually increased with the dimensionality in all analyses, highlighting the fact that the absolute magnitude of CCC does not unbiasedly reflect the actual relationship between brain imaging measures and subject measures especially when the SVR is low.

### 4.1 The stability of CCA results is affected by the SVR and the correlation strength between two sets of variables

We found that the stability of CCA results changed mainly as a function of the SVR (i.e., the data dimensionality when the sample size is fixed) and the correlation strength between the two sets of variables of interest (here, the brain imaging measures and the subject measures).

Regarding the correlation strength between brain imaging measures and subject measures, we found that CCA results were generally stable (i.e., small differences in CCC, consistent statistical significance and strong correlation between loading vectors) for a range of higher SVRs (Tianjin data set: SVR > 1.34 for GMV and SVR > 1.56 for ReHo; HCP data set: SVR > 1.43) if there was a strong correlation between the two sets of variables (Tianjin data set: ~0.89 for GMV and ~0.82 for ReHo at a SVR of 9.36; HCP data set: ~0.85 for GMV at a SVR of 10) but became clearly instable (i.e., inconsistent statistical significance and/or weak correlation between loading vectors) even for very high SVRs (i.e., >10) if there was only a moderate correlation between the two sets of variables. This result was observed for all subject overlapping rates (even for the highest subject overlapping rate, that is, 450 of 468 subjects were identical between two subgroups in Tianjin data set; 315 of 350 subjects were identical between two subgroups in HCP data set), for both the structural imaging measures (i.e., GMV) and the functional imaging measures (ReHo), and in both data sets, indicating that CCA is recommended only when a strong relationship between the two sets of variables is expected. This finding is particularly important as the influence of the correlation strength on the stability of CCA results has been largely overlooked in previous studies. Indeed, many previous studies reported moderate CCCs—for example, 0.43 for a SVR of 8 (Lin et al., 2018) and 0.54 for a SVR of 9.97 (Rodrigue et al., 2018), or strong CCCs but at a relatively low SVR—for example, 0.87 for a SVR of 3.64 (Will et al., 2017) and 0.87 for a SVR of 4.61 (Smith et al., 2015), and the stability of their results may need to be reevaluated.

Regarding the SVR, we found that the stability of CCA results decreased with the increase of the SVR. This is expected and confirmed in both the “strong correlation” scenario and the “moderate correlation” scenario, for all subject overlapping rates, for both structural (i.e., GMV) and functional imaging measures (ReHo), and in both data sets. This result is also consistent with the previous findings obtained based on simulated data showing that the power of CCA to detect a significant canonical correlation decreased as the SVR decreased (Mendoza, Markos, & Gonter, 1978; Naylor, Lin, Weiss, Raby, & Lange, 2010). This issue is also known as the “curse of dimensionality” and manifested as an over‐fitting problem in this case where any direction can be fitted in at least one set of variables, resulting in a perfect canonical correlation between two sets of variables, when the number of variables is equal to or greater than the number of subjects. Therefore, for a given sample size (in practice, the available number of subjects is also the limiting factor), the number of variables which can be included in the CCA should be well below the sample size. Having said this, it is also problematic if the dimensionality is too low. For neuroimaging studies, the original dimensionality of the brain imaging data is usually the number of voxels (or connections) of interest and thus is far more than the number of subjects. Therefore, PCA is usually a necessary step to reduce the dimensionality of the brain imaging data before CCA. Consequently, the number of PCs that are kept determines the dimensionality of the brain imaging data. Obviously, if too few PCs are kept, they cannot represent well the original brain imaging data and thus the resultant CCC may not truthfully reflect the relationship between the brain imaging data and the behavioral data either. Therefore, the number of PCs that are kept for CCA should be as large as possible while maintaining the stability of the CCA results. For the two data sets used in the present study, in the “strong correlation” scenario, the CCA results remained stable for a wide range of relatively low dimensionalities and then became instable as the dimensionality further increased—as shown in Figures 2c,d and 3b–d and Figures 6c–e and 7b–d (left column), when the number of PCs in the set of brain imaging measures increased to 400 (i.e., SVR > 468/350 = 1.34) for GMV, to 350 (SVR > 468/300 = 1.56) for ReHo when using Tianjin data set and to 280 (SVR > 350/245 = 1.43) for GMV when using HCP data set, the CCA results became clearly instable, manifested as a clear reduction of the correlation coefficients between loading vectors and the appearance of inconsistent statistical significance. In contrast, in the “moderate correlation” scenario of two data sets, stable results were observed in none of these analyses. In practice, the number of PCs is usually determined by a predefined percentage of the variance of the original data that can be explained by how many PCs (i.e., if the predefined percentage of variance to be explained is 85% and 100 PCs explain greater than 85% but 99 PCs explain less than 85% of the original variance, 100 PCs will be kept for CCA). Our results suggest that both the proportion of the original variance explained and the SVR should be considered when determining how many PCs should be kept for CCA. The priority should be put on the stability of CCA results—if the number of PCs that are required for explaining at least 85% of the original variance is too large to ensure a stable CCA result, the stability of CCA results must be considered first and the representability of the PCs has to be compromised.

We also noticed that instable results could still occur even when the subjects included in two subgroups were mostly overlapped: even for the largest overlapping rate (i.e., 450 of 468 subjects were identical and only 16 subjects were different, that is, over 96% overlapping between two subgroups in Tianjin data set; 315 of 350 subjects were identical and only 35 subjects were different, that is, 90% overlapping between two subgroups in HCP data set), the CCA results were still instable—inconsistent statistical significance and/or very low correlation coefficients between loading vectors when the dimensionality was high (e.g., Figures 2c–e and 3b–d, 4c–e and 5b–d, 6c–e and 7b–d) or when the actual correlation between brain imaging measures and subject measures was only moderate (e.g., Figures 4c–e and 5b–d, the right column of Figures 6c–e and 7b–d). This observation suggests that, if the stability requirements were not met (i.e., insufficient SVR or correlation strength), even just a slight change of subjects in the data set to be analyzed could change greatly the CCA results.

In addition to the above assessments of CCA stability which were based on the similarity of the CCA results obtained from two independent CCAs performed in two subgroups, we also assessed the CCA stability using a different strategy—we directly applied the canonical weights (i.e., the transformation vectors *A1*/*B1*, see Methods section) obtained from a discovery data to a held‐out data and tested whether the statistical significance of the correlation between the resultant pseudocanonical variables of the held‐out data were consistent with that of the discovery data. The detailed analysis procedure is provided in the Supplemental Methods (Additional Analysis 2) and the detailed results are shown in Figure S24. In brief, after removing the effects of PC inconsistency between the discovery data and the held‐out data, the results of this test confirmed again that the CCA results were stable only when the SVR was sufficiently high and the canonical correlation was sufficiently strong. Our results also showed that the CCA results (i.e., the resultant CCCs and their statistical significance) obtained from the discovery data and the held‐out data were highly inconsistent even for high SVRs in the “strong correlation” scenario if the PCA were performed separately in each subgroup, suggesting that, when applying identical canonical weights to the discovery data and the held‐out data, the inconsistency between the PCs obtained separately from the two data sets (i.e., two groups of subjects) could also be a major source of the instability of CCA results.

Furthermore, it is worth mentioning that, although only the first CCA mode was tested in the present study to avoid excessively complicated analyses, it seems reasonable to infer that the stability of the subsequent CCA modes (i.e., *U2*/*V2*, *U3*/*V3*, etc.) would be considerably worse based on the current results—the stability of CCA results is strongly dependent on the correlation strength. Indeed, the CCCs of the second or the third mode would be smaller than that of the first mode by design (i.e., CCC1 > CCC2 > CCC3), and thus all subsequent pairs of canonical variables are less likely to be stable compared with the first pair. Similarly, we did not include a “weak correlation” scenario in the present study because it is expected that the CCA results would be instable when the two sets of variables are only weakly correlated.

### 4.2 CCA does not offer a truthful measure of the correlation between brain imaging data and behavioral data

Although CCC, as designed, can measure the correlation between two sets of variables to some degree as confirmed by the reduction of CCC magnitude after removing three subject measures which had strong correlations with brain imaging measures, our results also highlight the fact that the CCC should not be regarded as a truthful measurement of the relationship between brain imaging data and subject data. Indeed, our results clearly showed that the magnitude of CCC always increased with the data dimensionality and it was always relative to a particular SVR. This is theoretically well established—as mentioned before, the first‐mode CCC is the maximal correlation of linear combinations of variables between two sets of variables, and thus the first‐mode CCC will inevitably reach 1 when the number of variables reaches the number of subjects resulting in a full‐rank space (i.e., the set of variables with a full rank can compose any direction in alignment with a particular direction identified in the other set of variables). Therefore, the CCC alone is not interpretable—the CCC should always be considered and reported together with the corresponding SVR. However, this issue is sometimes overlooked in previous studies where CCCs were interpreted as strong or weak without considering the SVRs (Davis et al., 2011; Kottaram et al., 2019; Lin et al., 2018; Rodrigue et al., 2018; Smith et al., 2015; Will et al., 2017). Our results suggest that the absolute magnitudes of CCCs should be interpreted with extra caution and are not directly comparable between different studies without properly considering the SVR.

### 4.3 Guidelines for applying CCA in neuroimaging studies to investigate the brain‐behavior relationship

Based on the findings discussed above, we provide the following guidelines if one plans to apply CCA in neuroimaging studies to investigate the relationship between brain imaging measures and subject measures:

- The stability of CCA results is not guaranteed and thus CCA is not recommended if the correlation between brain imaging measures and subject measures is not expected to be strong. In practice, we recommend to take the SVR of 9 as a reference by selecting the appropriate number of PCs for a given sample size and then check the corresponding CCC—if the resultant CCC is less than 0.65, the stability of the CCA results cannot be guaranteed.
- If the above step suggests a strong correlation between brain imaging measures and subject measures (i.e., CCC > 0.8 when SVR = 9), the number of variables (i.e., the dimensionality) in each variable set should not be higher than two thirds (or even half, to be safer) of the sample size as the stability of the CCA results is not guaranteed otherwise. In practice, for a given sample size, if the number of the original variables does not meet this criterion, it is recommended to perform PCA on the original variable set and select the appropriate number of PCs which can explain as much original variance as possible.
- The SVR (or equivalent information such as the dimensionality of each data set and the sample size) should be reported alongside with the CCC. Interpreting the magnitude of CCC as an absolute measure of the correlation between brain imaging measures and subject measures should be avoided.

### 4.4 Limitations

There are several limitations to the present study. First, our assessment of the stability of CCA results was only focused on the first mode to avoid excessively complicated analyses and also because it is the most important and the most commonly reported results in CCA studies. Although it is plausible to expect that the same principles of CCA stability obtained from the first mode described above should also apply to other modes, confirmation is needed in future studies. Second, in order to reduce the computational load—one CCA need to be performed for each of the 2,000 subgroups (i.e., 1,000 pairs of subgroups) for each of the 720 combinations (9 SVRs × 10 subject overlapping rates × 2 types of brain imaging measures [i.e., GMV and ReHo] × 2 correlation strengths scenarios × 2 procedures [i.e., main procedure and control procedure] = 720), leading to 1.44 × 10^6^ CCAs in total for just the Tianjin data set, our assessment of the statistical significance of each first‐mode CCC was determined by a relatively small number of permutation tests (*n* = 100). Although it is unlikely that the observed instable results in the “moderate correlation” scenario were due to a low number of permutations as similar results were obtained even using a much larger number of permutations (*n* = 10,000; Figure S7), a higher number of permutations (*n* > 1,000) is recommended in a real CCA application. Third, a few modified versions of the classical CCA, such as kernel CCA (Hardoon et al., 2004) and sparse CCA (Witten, Tibshirani, & Hastie, 2009), have been proposed. Specifically, Kernel CCA is designed to capture the complicated nonlinear relationship between two sets of variables by mapping the original feature space into a new feature space through a predefined kernel function. Sparse CCA induces sparsity on canonical coefficients by imposing the *L*_1_‐norm penalty and therefore it can be used to deal with high‐dimensional variables. Future work is needed to test whether they behave differently in terms of stability in applications in neuroimaging studies.

## 5 CONCLUSIONS

Although CCA is a promising multivariate approach which holds many advantages in exploring the relationship between the human brain and behavior, CCA cannot be used without restriction. The results of our systematic investigation of CCA stability in the context of neuroimaging, replicated in both structural and functional imaging measures and in two independent data sets, showed that two requirements need to be met at the same time to ensure the stability of CCA results: a sufficiently high SVR and a sufficiently strong correlation between the investigated brain imaging measures and subject measures. Importantly, we characterized the CCA stability quantitatively from three aspects using a series of SVRs in different correlation strength scenarios and provided a guideline for proper applications of CCA in neuroimaging studies based on our findings.

## CONFLICT OF INTEREST

The authors declare no competing financial interests.

## ETHICS STATEMENT

Ethical approval of the Tianjin Dataset was obtained from the medical ethics committee of Tianjin Medical University General Hospital and the medical ethics committee of Tianjin Medical University Cancer Institute and Hospital prior to the study and written informed consent was obtained from each participant before enrollment.

## Supporting information


**Appendix S1** Supporting Information.

## Data Availability

This study involved two large data sets (Tianjin Dataset and HCP Dataset). The HCP Dataset is publicly available at https://db.humanconnectome.org. The Tianjin Dataset is part of the CHIMGEN Study and available through applications to the committee of the CHIMGEN Study (http://chimgen.tmu.edu.cn; chunshuiyu@tmu.edu.cn). The codes supporting the findings of this study are available from the corresponding author upon request.
