# Supplementary material for: Stability test of canonical correlation analysis for studying brain‐behavior relationships: The effects of subject‐to‐variable ratios and correlation strengths
Source: Hum Brain Mapp. 2021 Feb 24;42(8):2374–92. doi: 10.1002/hbm.25373 (PMC8090773; doi:10.1002/hbm.25373)
Supplement: Supplementary file 1 — Appendix S1 Supporting Information. [file HBM-42-2374-s001.docx]

**Supplemental information**

for

Stability test of canonical correlation analysis for studying brain-behavior relationships: The effects of subject-to-variable ratios and correlation strengths

*Qingqing Yang, Xinxin Zhang, Yingchao Song, Feng Liu, Wen Qin, Chunshui Yu, Meng Liang*

Supplemental Methods

Additional Analysis 1: testing the consistency of the significance of the voxel loadings between paired subgroups

In addition to the correlation coefficients of the loading vectors between paired subgroups, we also examined the consistency of the significance of the voxel loadings between paired subgroups to test whether the same voxels (i.e., brain areas) were detected to have significant loadings in both subgroups of each pair. This additional test was performed using the GMV data of the Tianjin data set for each SVR in both the “strong correlation” scenario and the “moderate correlation” scenario. The detailed procedure is as follows: (1) The whole data set (n=936) was randomly split into two subgroups (n=468 for each subgroup) with 0 overlapping subjects. (2) For each subgroup, PCA was applied to imaging data (the number of kept PCs ranged from 50 to 450 with an increment of 50) and also to the subject measure data (the number of kept PCs was fixed to 50). (3) For each subgroup, CCA was performed between the imaging data and the subject measure data using the kept PCs and then the loadings of all imaging variables (i.e., all voxels) for the first mode were calculated. (4) For each subgroup, bootstrap testing (n=100) was performed to estimate the confidence interval (CI) for each voxel loading – if the 95% CI of a voxel loading did not include zero, the loading of this voxel was considered significant and robust. (5) The above procedure was repeated 100 times, each with a random split of the whole data set into two paired subgroups, resulting in a percentage for each voxel of being consistently significant in both subgroups over all pairs.

Additional Analysis 2: testing the significance of the correlation between the canonical variables identified from a discovery data in a held-out data

In this additional analysis, we assessed the CCA stability using a different strategy – we directly applied the canonical weights (i.e., the transformation vectors *A1*/*B1*, see Methods section) obtained from a discovery data to a held-out data and tested whether the statistical significance of the correlation between the resultant pseudo canonical variables of the held-out data was consistent with that of the discovery data. This analysis was performed using the GMV data of the Tianjin data set for each SVR in both the “strong correlation” scenario and the “moderate correlation” scenario, with or without removing the PC inconsistency.

The detailed analysis procedure without removing the PC inconsistency between the discovery data and the held-out data is as follows: (1) The whole Tianjin data set (n=936; GMV data) was randomly split into two subgroups (n=468 for each subgroup) with 0 overlapping subjects – taking one subgroup as discovery data and the other as held-out data. (2) For each subgroup, PCA was applied to the imaging data (the number of kept PCs ranged from 50 to 450 with an increment of 50) and also to the subject measure data (the number of kept PCs was fixed to 50). (3) For the discovery data, CCA was performed between imaging measures and subject measures using the kept PCs – alongside the first-mode CCC, its statistical significance determined using permutation tests (n=100; CCC was considered to be significant when p<0.05) and the canonical variables *U1* and *V1*, the corresponding combination weights of each PC were also obtained. (4) The combination weights of the first CCA mode obtained from the discovery data (i.e., from the third step) were directly applied to the PCs of the held-out data (i.e., a linear combination of the PCs of the held-out data with the weights obtained from the discovery data) to obtain the pseudo canonical variable scores *U1’* and *V1’* of the held-out data. (5) The Pearson correlation coefficient was calculated between *U1’* and *V1’* as the pseudo CCC’ of the held-out data and its statistical significance was determined using permutation tests (n=100; CCC was considered to be significant when p<0.05). (6) The above steps were repeated 1,000 times in the “strong correlation” scenario and also in the “moderate correlation” scenario. The results are summarized in Supplemental Figure S24A&B.

The analysis procedure with the PC inconsistency between the discovery data and the held-out data removed (i.e., to ensure that the PCs used in the two data were entirely corresponding to each other) was the same as the one described in the previous paragraph except for Step (2) and Step (4): In Step (2), PCA was only performed in the discovery data to obtain a transformation matrix which projects the original variables into a series of PCs for the imaging/subject measures, and then the same transformation matrix obtained from the discovery data was applied to the original variables of the held-out data to derive the pseudo PCs of the held-out data, and thus these pseudo PCs of the held-out data correspond perfectly with the PCs of the discovery data. In Step (4), these pseudo PCs of the held-out data and the combination weights of the first CCA mode obtained from the discovery data were used to derive the pseudo canonical variable scores *U1’* and *V1’* of the held-out data. The results are summarized in Supplemental Figure S24A&C.

Supplemental Figures

**
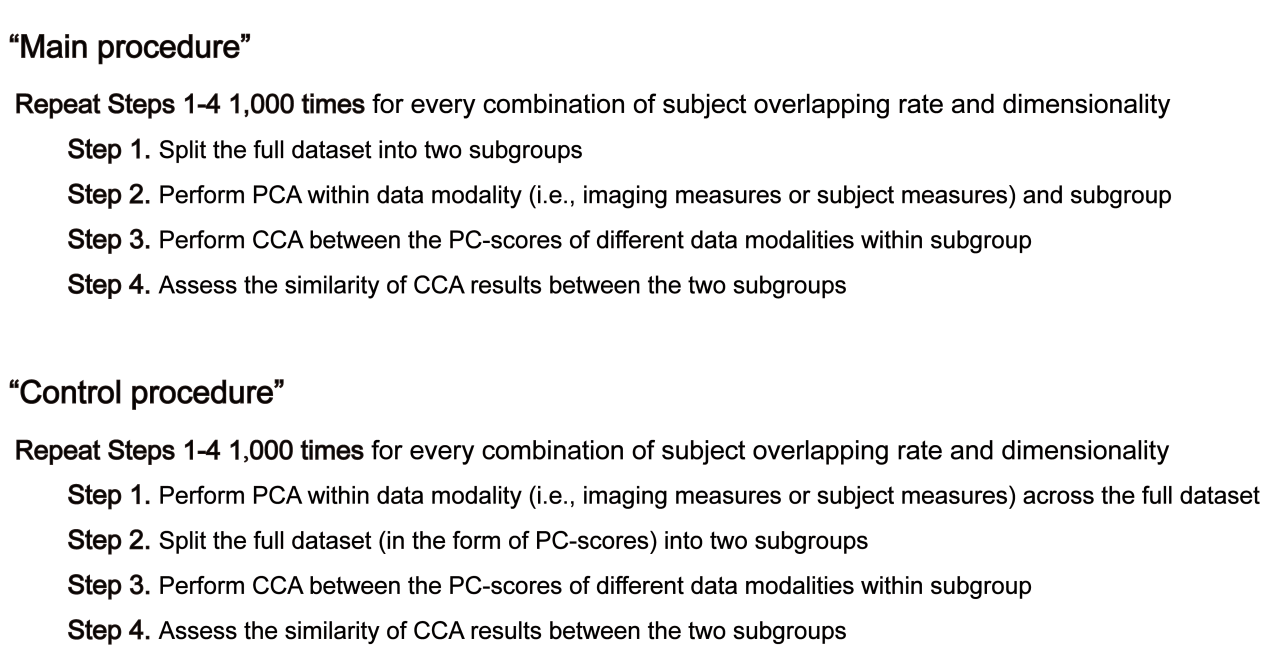
**

**Figure S1.** The pseudo-code of the “main procedure” and the “control procedure”.

**
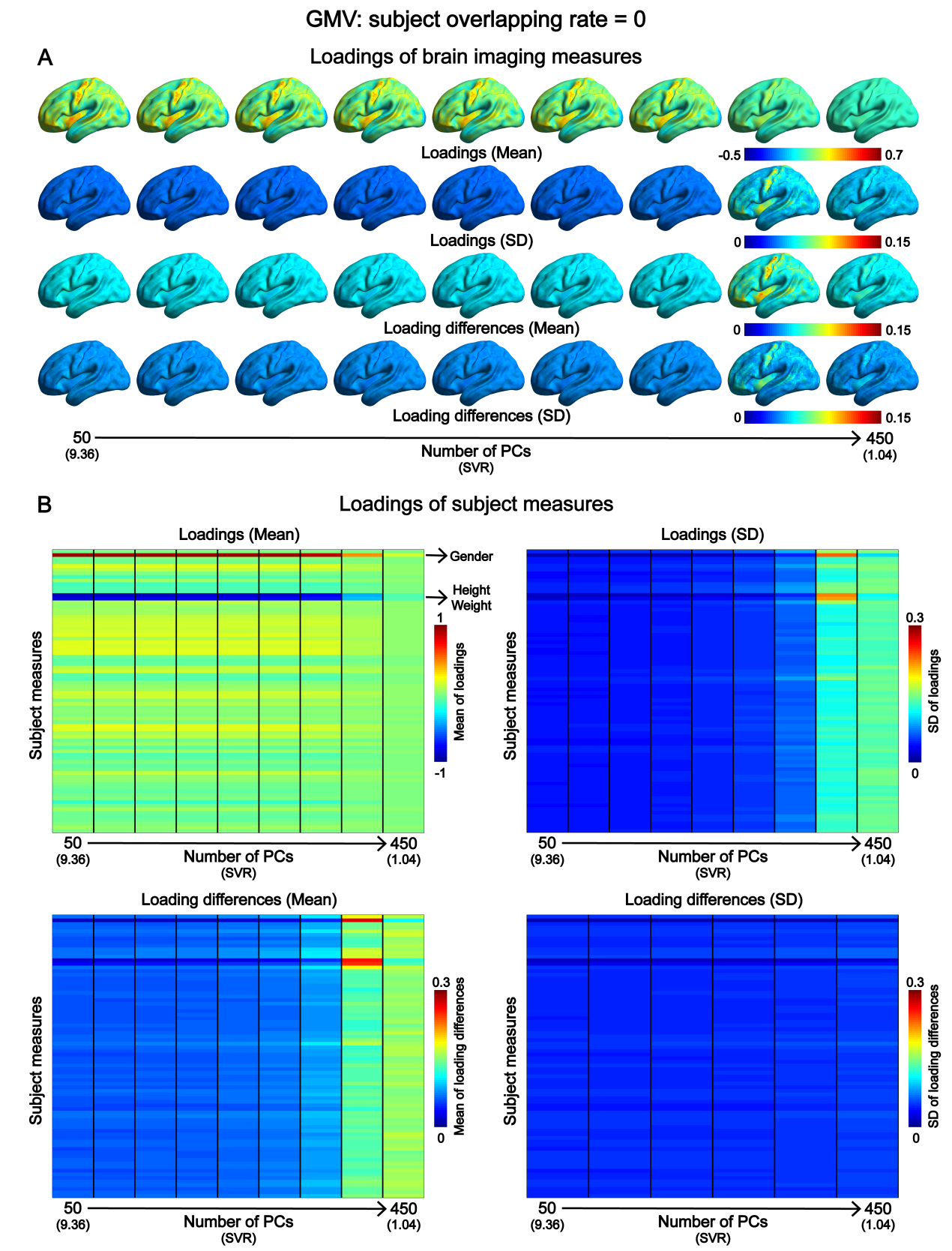
**

**Figure S2.** The results of loadings of brain imaging measures (panel A) and subject measures (panel B) of CCA between GMV and subject measures when there are no overlapping subjects between the two subgroups of 1,000 pairs of CCAs using Tianjin data set in the “main procedure” with all 78 subject measures (i.e., the “strong correlation” scenario). The mean and the standard deviation (SD) of the loadings across 2,000 CCAs are shown in the upper two rows in Panel A for brain imaging measures and in the upper part of Panel B for subject measures. The mean and the SD of the absolute differences in loadings between two paired subgroups of 1,000 pairs are shown in the lower two rows in Panel A for brain imaging measures and in the lower part of Panel B for subject measures. In panel A, loadings are arranged in brain space and for all dimensionalities of imaging measures ranging from 50 to 450 with a step of 50 (increasing from left to right) and the corresponding SVRs ranging from 9.36 to 1.04 (decreasing from left to right). In panel B, loadings are arranged in the form of a matrix with rows indicating variables and columns indicating dimensionalities and the corresponding SVRs.


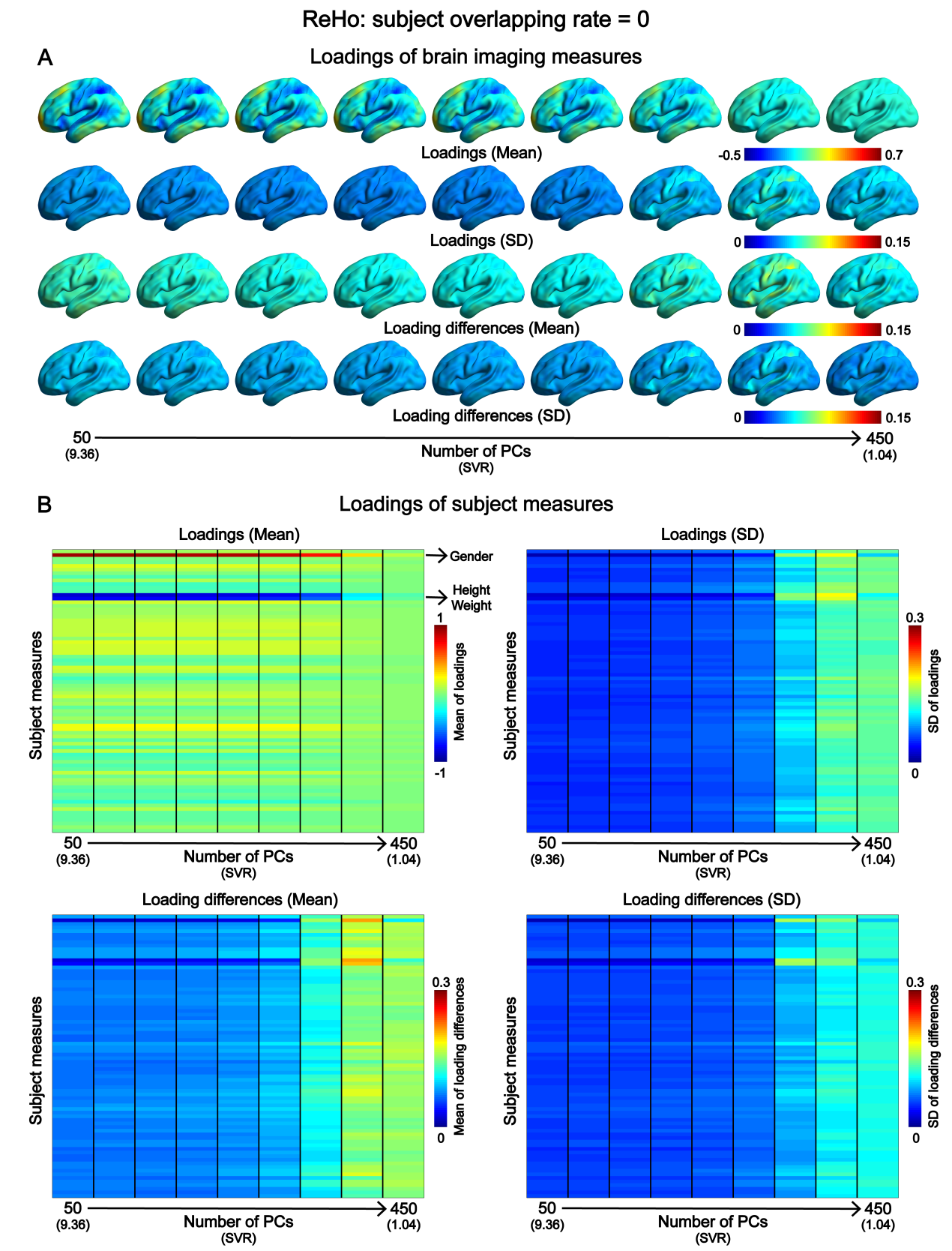


**Figure S3.** The results of loadings of brain imaging measures (panel A) and subject measures (panel B) of CCA between ReHo and subject measures when there are no overlapping subjects between the two subgroups of 1,000 pairs of CCAs using Tianjin data set in the “main procedure” with all 78 subject measures (i.e., the “strong correlation” scenario). The mean and the standard deviation (SD) of the loadings across 2,000 CCAs are shown in the upper two rows in Panel A for brain imaging measures and in the upper part of Panel B for subject measures. The mean and the SD of the absolute differences in loadings between two paired subgroups of 1,000 pairs are shown in the lower two rows in Panel A for brain imaging measures and in the lower part of Panel B for subject measures. In panel A, loadings are arranged in brain space and for all dimensionalities of imaging measures ranging from 50 to 450 with a step of 50 (increasing from left to right) and the corresponding SVRs ranging from 9.36 to 1.04 (decreasing from left to right). In panel B, loadings are arranged in the form of a matrix with rows indicating variables and columns indicating dimensionalities and the corresponding SVRs.


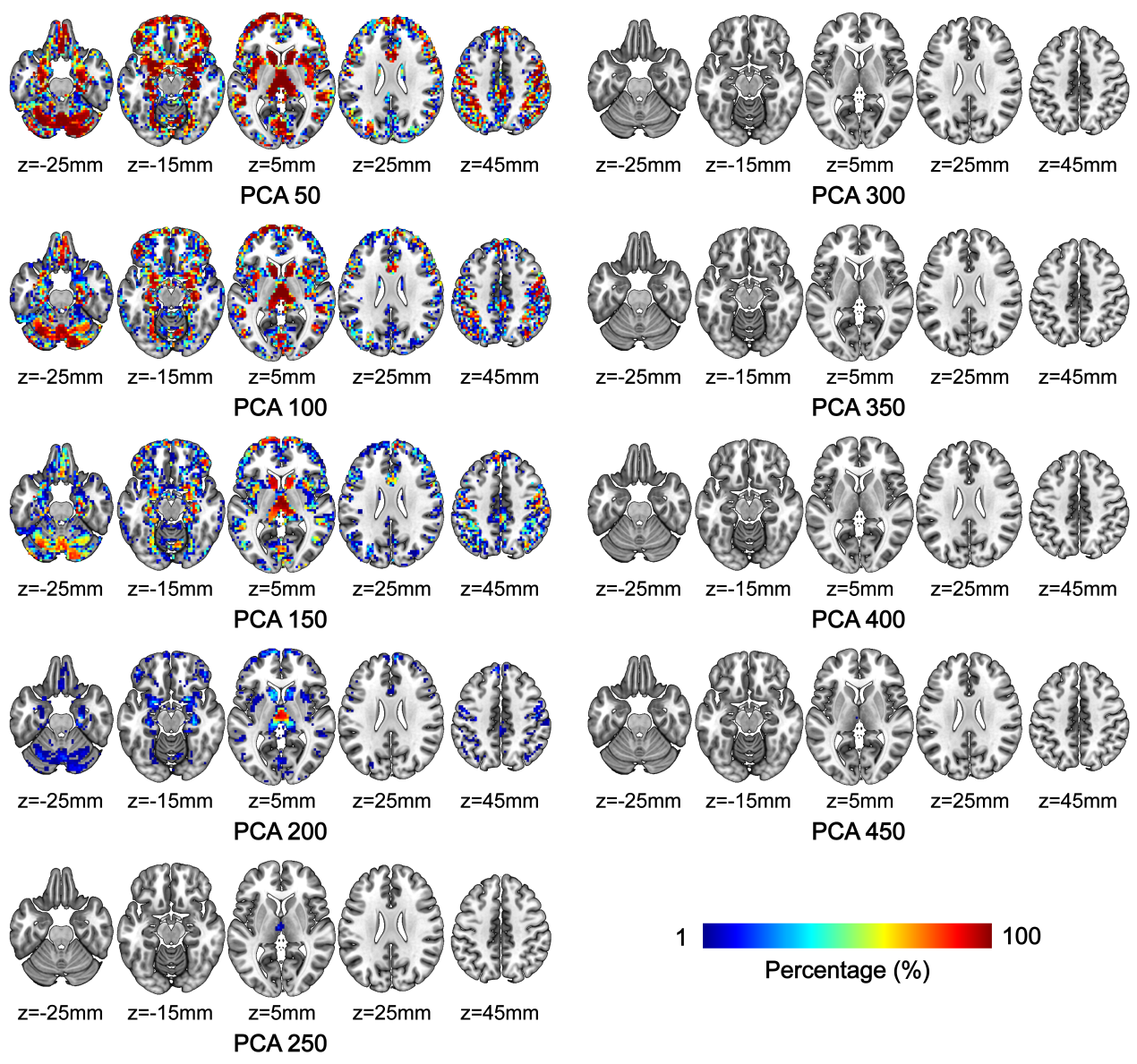


**Figure S4.** Brain maps showing the frequency for each voxel of being detected to have consistently robust loadings in two paired subgroups over 100 pairs in the “strong correlation” scenario for each dimensionality in the brain imaging domain (i.e., the number of kept PCs; ranging from 50 to 450 with an increment of 50) obtained using the Tianjin data set. The frequency is color-coded between 1 (dark blue, indicating that the voxel was detected to have robust loadings in both subgroups in only one out of 100 pairs) and 100 (dark red, indicating that the voxel was detected in all 100 pairs).

**
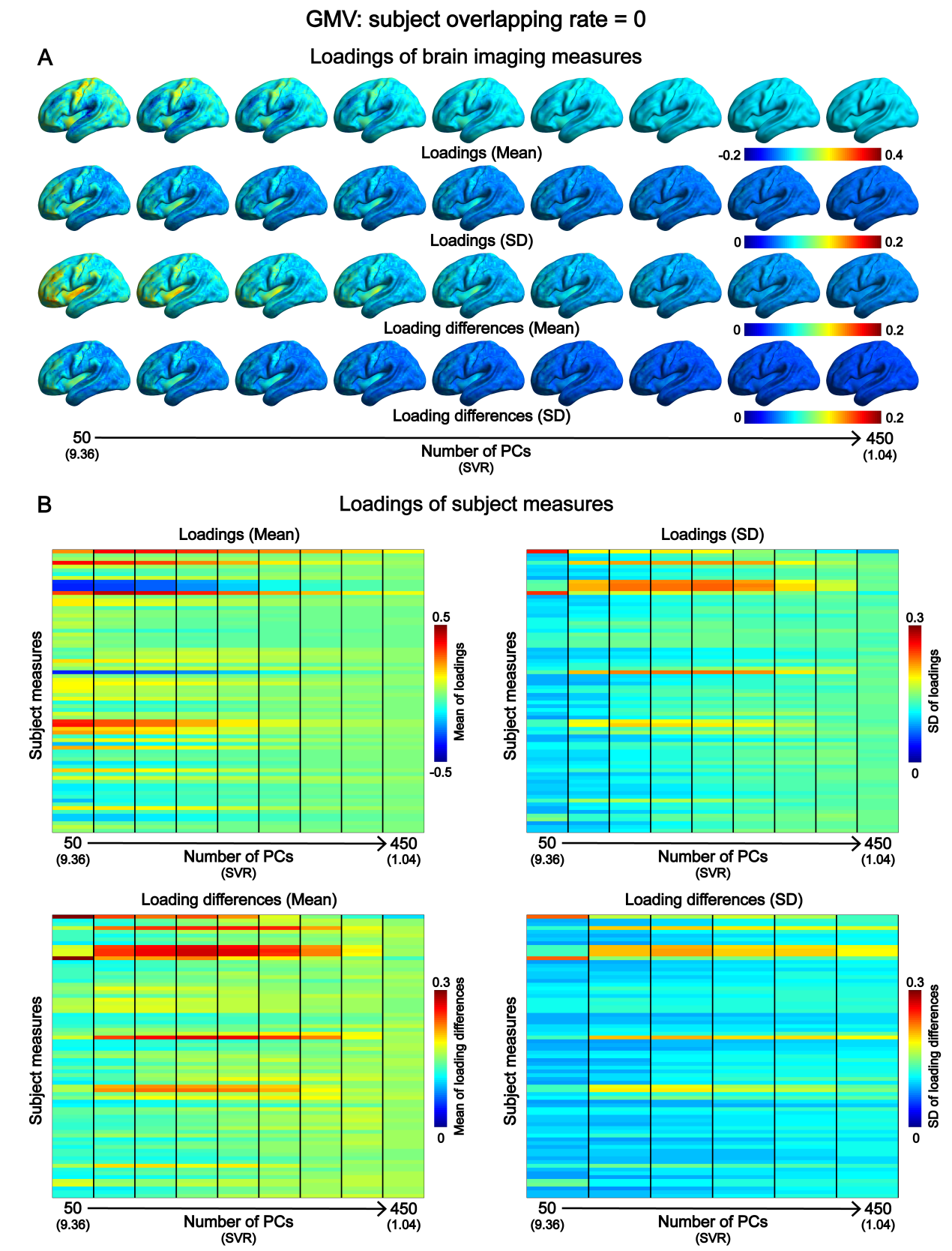
**

**Figure S5.** The results of loadings of brain imaging measures (panel A) and subject measures (panel B) of CCA between GMV and subject measures when there are no overlapping subjects between the two subgroups of 1,000 pairs of CCAs using Tianjin data set in the “main procedure” with 75 subject measures (i.e., the “moderate correlation” scenario). The mean and the standard deviation (SD) of the loadings across 2,000 CCAs are shown in the upper two rows in Panel A for brain imaging measures and in the upper part of Panel B for subject measures. The mean and the SD of the absolute differences in loadings between two paired subgroups of 1,000 pairs are shown in the lower two rows in Panel A for brain imaging measures and in the lower part of Panel B for subject measures. In panel A, loadings are arranged in brain space and for all dimensionalities of imaging measures ranging from 50 to 450 with a step of 50 (increasing from left to right) and the corresponding SVRs ranging from 9.36 to 1.04 (decreasing from left to right). In panel B, loadings are arranged in the form of a matrix with rows indicating variables and columns indicating dimensionalities and the corresponding SVRs.

**
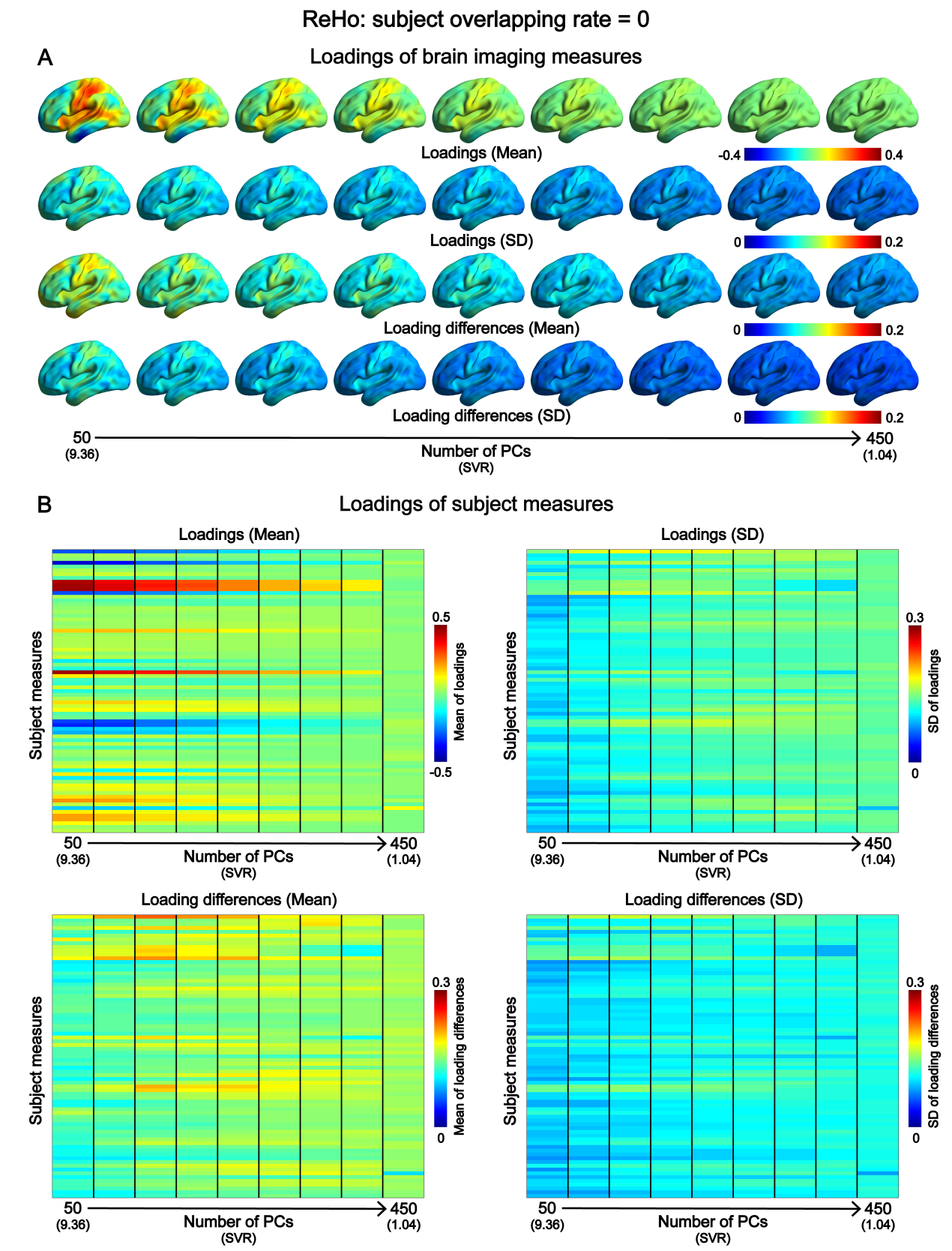
**

**Figure S6.** The results of loadings of brain imaging measures (panel A) and subject measures (panel B) of CCA between ReHo and subject measures when there are no overlapping subjects between the two subgroups of 1,000 pairs of CCAs using Tianjin data set in the “main procedure” with 75 subject measures (i.e., the “moderate correlation” scenario). The mean and the standard deviation (SD) of the loadings across 2,000 CCAs are shown in the upper two rows in Panel A for brain imaging measures and in the upper part of Panel B for subject measures. The mean and the SD of the absolute differences in loadings between two paired subgroups of 1,000 pairs are shown in the lower two rows in Panel A for brain imaging measures and in the lower part of Panel B for subject measures. In panel A, loadings are arranged in brain space and for all dimensionalities of imaging measures ranging from 50 to 450 with a step of 50 (increasing from left to right) and the corresponding SVRs ranging from 9.36 to 1.04 (decreasing from left to right). In panel B, loadings are arranged in the form of a matrix with rows indicating variables and columns indicating dimensionalities and the corresponding SVRs.


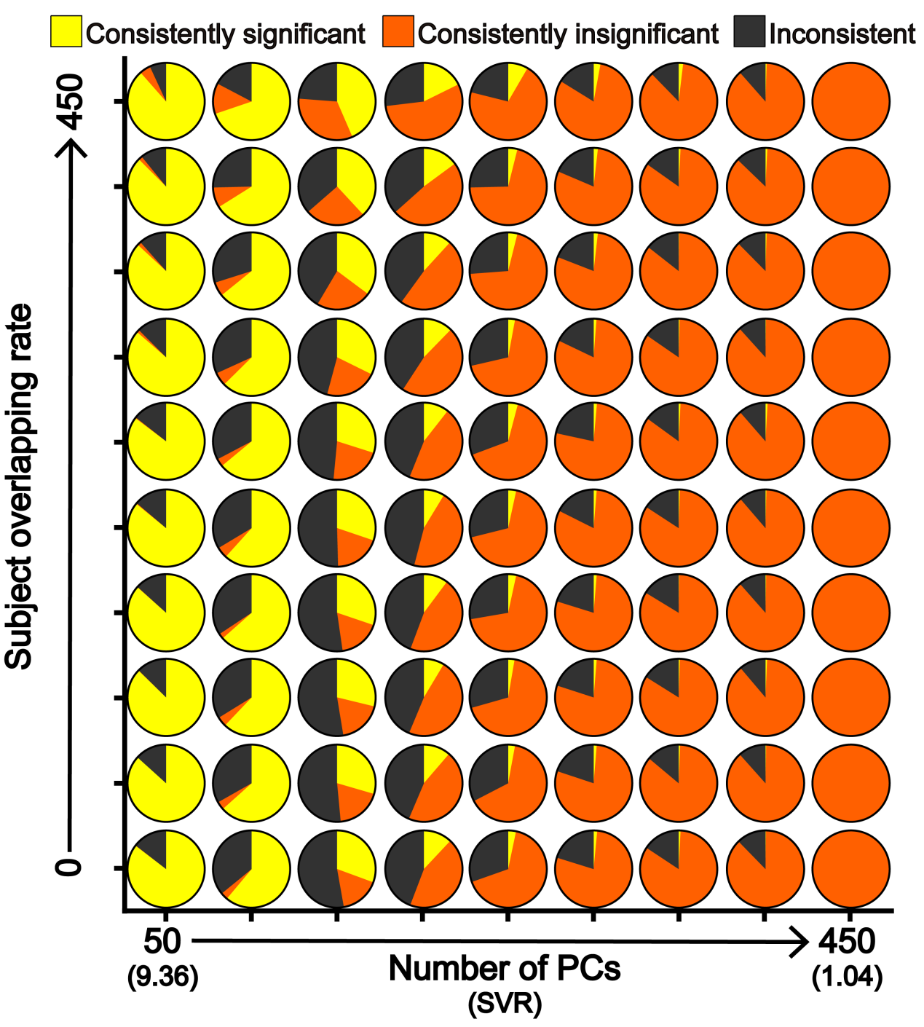


**Figure S7.** The results of the consistency of the statistical significance of CCCs between two subgroups of 1,000 pairs of CCAs for GMV using Tianjin data set in the “moderate correlation” scenario of the “main procedure” obtained using 10,000 permutations. This result was very similar to the result obtained using 100 permutations (left column of Fig. 4C). The x-axis represents the dimensionality of imaging measures (i.e., the number of kept PCs after PCA, ranging from 50 to 450 with a step of 50) and the corresponding SVR (i.e., the ratio of the sample size to the dimensionality of the imaging measures, ranging from 9.36 to 1.04). The y-axis represents the subject overlapping rates between two subgroups of each pair (ranging from 0 to 450 with a step of 50).

**
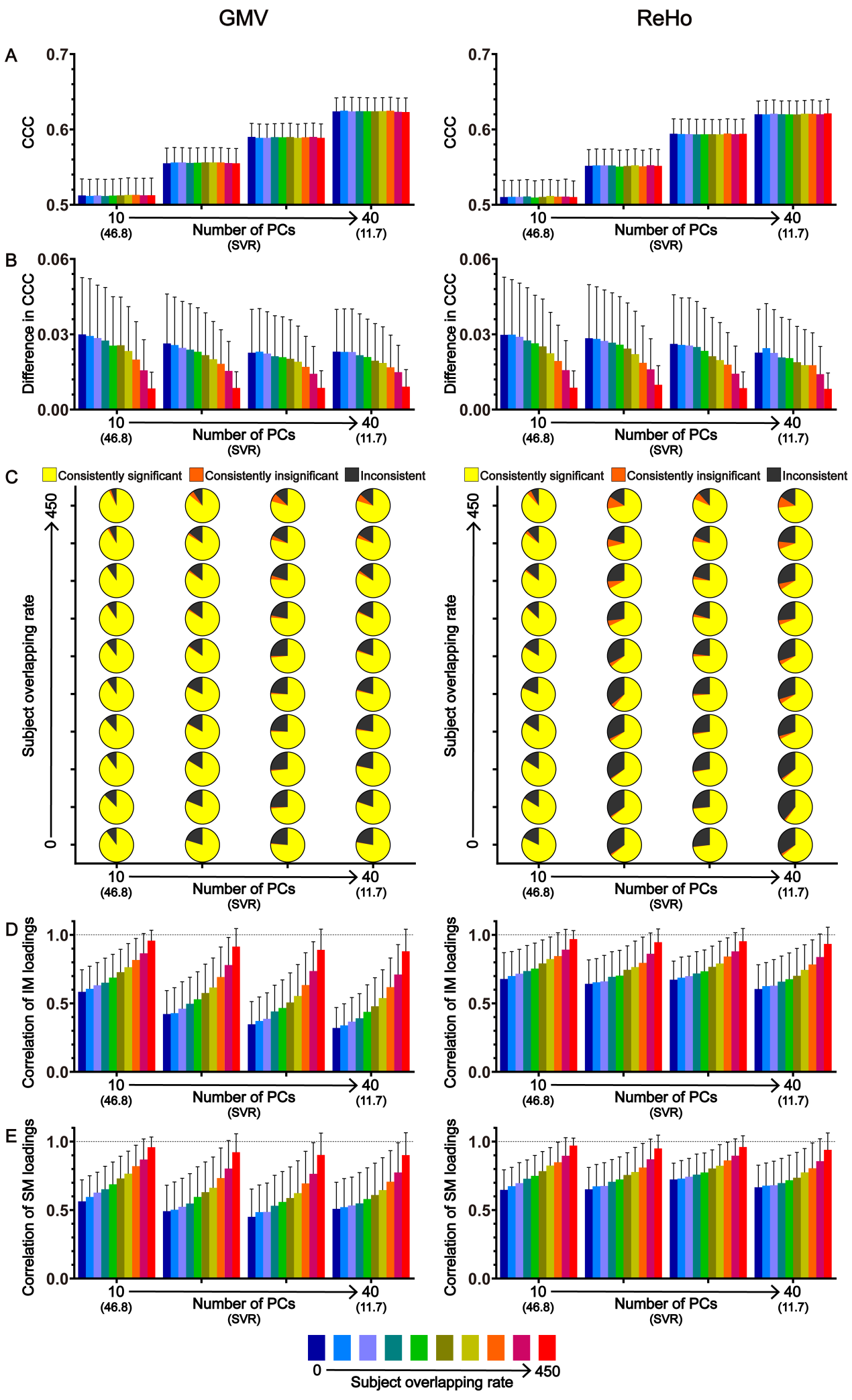
**

**Figure S8.** The results of CCA stability assessments using the Tianjin data set in the “main procedure” with 75 subject measures (i.e., the “moderate correlation” scenario). Panel A shows the magnitudes of CCCs obtained from 2,000 CCAs for all combinations of subject overlapping rate and data dimensionality. Panel B shows the absolute differences in CCCs of 1,000 pairs of CCA. Panel C shows the consistency of the statistical significance of CCCs between two subgroups of 1,000 pairs of CCAs. Panels D and E show the correlation coefficients of the loading vectors between two subgroups of 1,000 pairs of CCAs corresponding to brain imaging measures and those corresponding to subject measures, respectively. The abscissa of all subgraphs represents the dimensionality of imaging measures (i.e., the number of kept PCs after PCA, ranging from 10 to 40 with a step of 10) and the corresponding SVR (i.e., the ratio of the sample size to the dimensionality of the imaging measures, ranging from 46.8 to 11.7). The subject overlapping rates between two subgroups of each pair (ranging from 0 to 450 with a step of 50) are color coded. In all bar plots, the height of the bars indicates the mean and the error bars indicate the standard deviation.

**
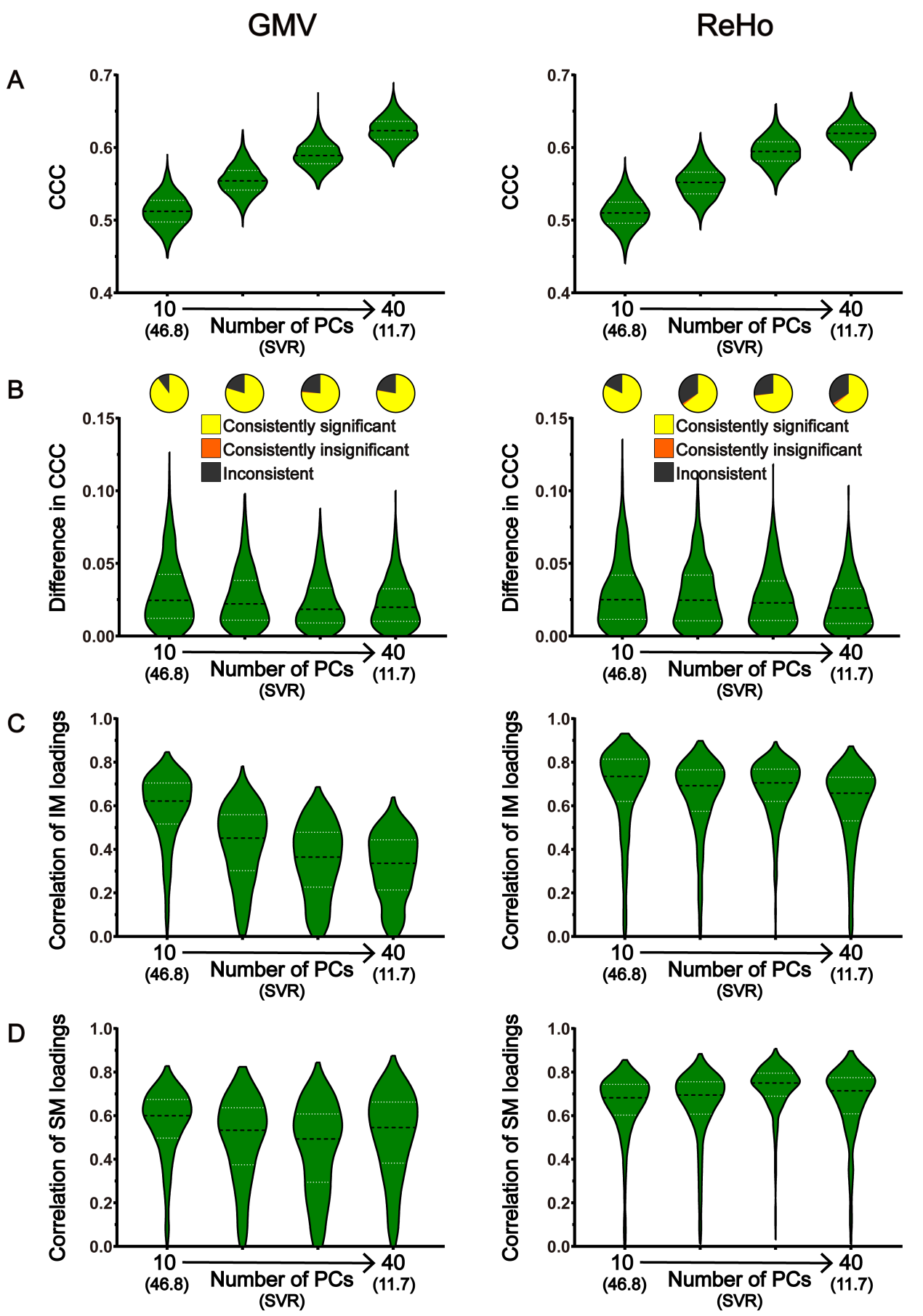
**

**Figure S9.** The results of CCA stability assessments using the Tianjin data set in the “main procedure” with 75 subject measures (i.e., the “moderate correlation” scenario) when there are no overlapping subjects between the two subgroups of 1,000 pairs of CCAs. Panel A shows the magnitudes of CCCs obtained from 2,000 CCAs for all data dimensionalities. Panel B shows the absolute differences in CCCs of 1,000 pairs of CCA (lower part) and the consistency of the statistical significance of CCCs between two subgroups of 1,000 pairs of CCAs (upper part). Panels C and D show the correlation coefficients of the loading vectors between two subgroups of 1,000 pairs of CCAs corresponding to brain imaging measures and those corresponding to subject measures, respectively. The abscissa of all subgraphs represents the dimensionality of imaging measures (i.e., the number of kept PCs, ranging from 10 to 40 with a step of 10) and the corresponding SVR (i.e., the ratio of the sample size to the dimensionality of the imaging measures, ranging from 46.8 to 11.7). In all smoothed violin plots, the sample median (black dotted line) and quartiles (white dotted line) are superimposed.

**
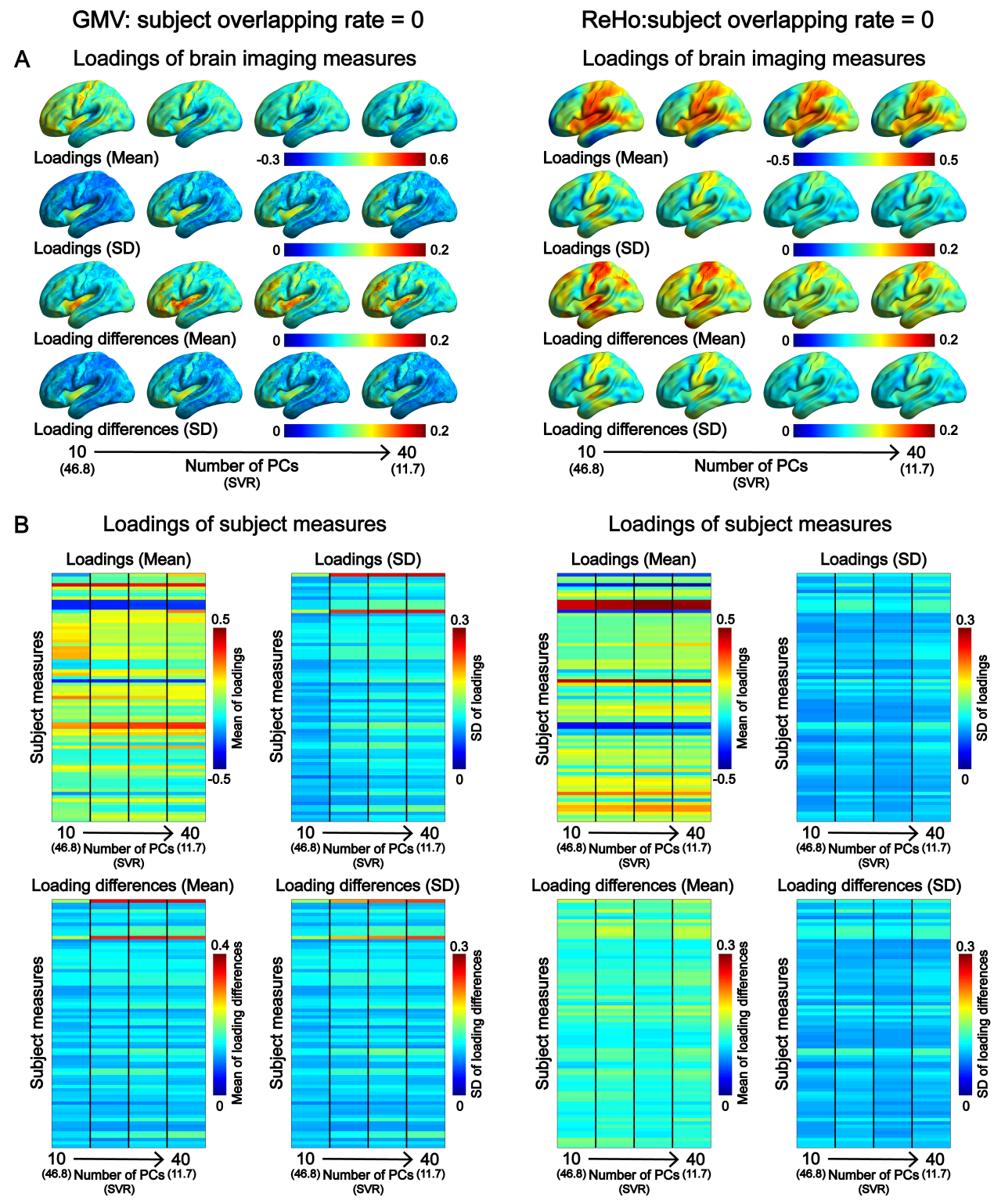
**

**Figure S10.** The results of loadings of brain imaging measures and subject measures of CCA between GMV and subject measures (left panel) and between ReHo and subject measures (right panel) for extremely low dimensionalities (i.e., 10, 20, 30 and 40) when there are no overlapping subjects between the two subgroups of 1,000 pairs of CCAs using Tianjin data set in the “main procedure” with 75 subject measures (i.e., the “moderate correlation” scenario). The mean and the standard deviation (SD) of the loadings across 2,000 CCAs are shown in the upper two rows in Panel A for brain imaging measures and in the upper part of Panel B for subject measures. The mean and the SD of the absolute differences in loadings between two paired subgroups of 1,000 pairs are shown in the lower two rows in Panel A for brain imaging measures and in the lower part of Panel B for subject measures. In panel A, loadings are arranged in brain space and for extremely low dimensionalities of imaging measures ranging from 10 to 40 with a step of 10 (increasing from left to right) and the corresponding SVRs ranging from 46.8 to 11.7 (decreasing from left to right). In panel B, loadings are arranged in the form of a matrix with rows indicating variables and columns indicating dimensionalities and the corresponding SVRs.

**
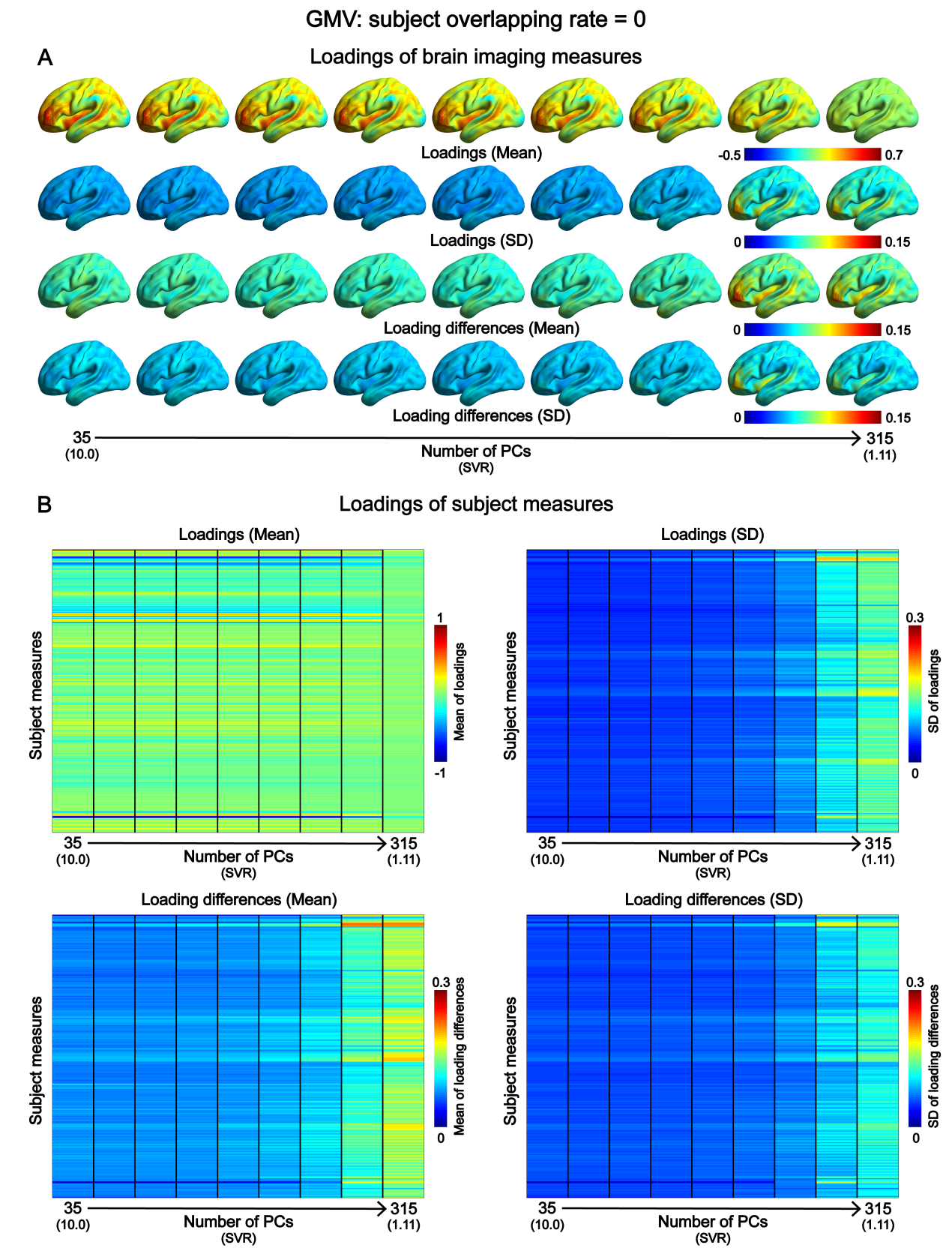
**

**Figure S11.** The results of loadings of brain imaging measures (panel A) and subject measures (panel B) of CCA between GMV and subject measures when there are no overlapping subjects between the two subgroups of 1,000 pairs of CCAs using HCP data set in the “main procedure” with all 290 subject measures (i.e., the “strong correlation” scenario). The mean and the standard deviation (SD) of the loadings across 2,000 CCAs are shown in the upper two rows in Panel A for brain imaging measures and in the upper part of Panel B for subject measures. The mean and the SD of the absolute differences in loadings between two paired subgroups of 1,000 pairs are shown in the lower two rows in Panel A for brain imaging measures and in the lower part of Panel B for subject measures. In panel A, loadings are arranged in brain space and for all dimensionalities of imaging measures ranging from 35 to 315 with a step of 35 (increasing from left to right) and the corresponding SVRs ranging from 10.0 to 1.11 (decreasing from left to right). In panel B, loadings are arranged in the form of a matrix with rows indicating variables and columns indicating dimensionalities and the corresponding SVRs.

**
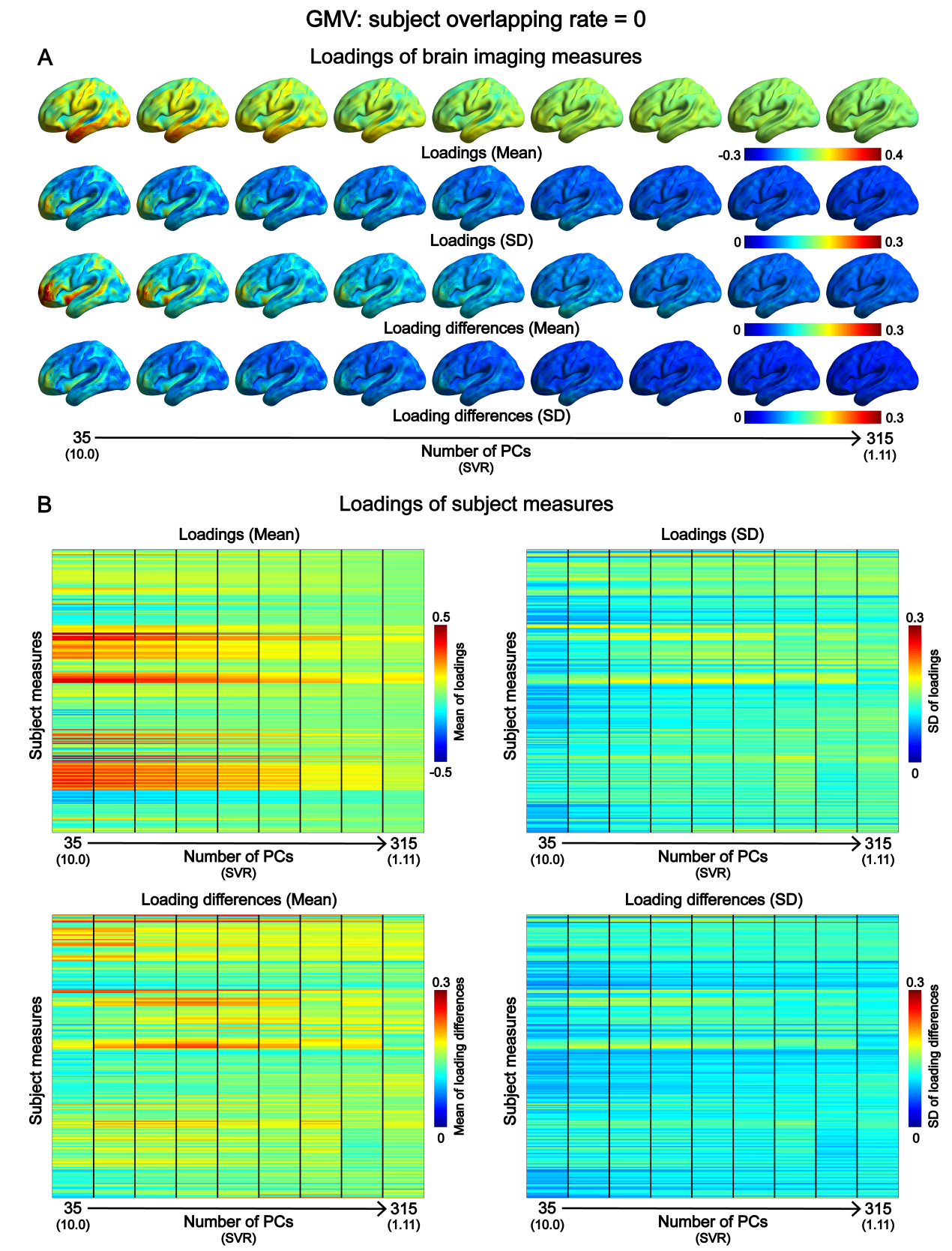
**

**Figure S12.** The results of loadings of brain imaging measures (panel A) and subject measures (panel B) of CCA between GMV and subject measures when there are no overlapping subjects between the two subgroups of 1,000 pairs of CCAs using HCP data set in the “main procedure” with 246 subject measures (i.e., the “moderate correlation” scenario). The mean and the standard deviation (SD) of the loadings across 2,000 CCAs are shown in the upper two rows in Panel A for brain imaging measures and in the upper part of Panel B for subject measures. The mean and the SD of the absolute differences in loadings between two paired subgroups of 1,000 pairs are shown in the lower two rows in Panel A for brain imaging measures and in the lower part of Panel B for subject measures. In panel A, loadings are arranged in brain space and for all dimensionalities of imaging measures ranging from 35 to 315 with a step of 35 (increasing from left to right) and the corresponding SVRs ranging from 10.0 to 1.11 (decreasing from left to right). In panel B, loadings are arranged in the form of a matrix with rows indicating variables and columns indicating dimensionalities and the corresponding SVRs.

**
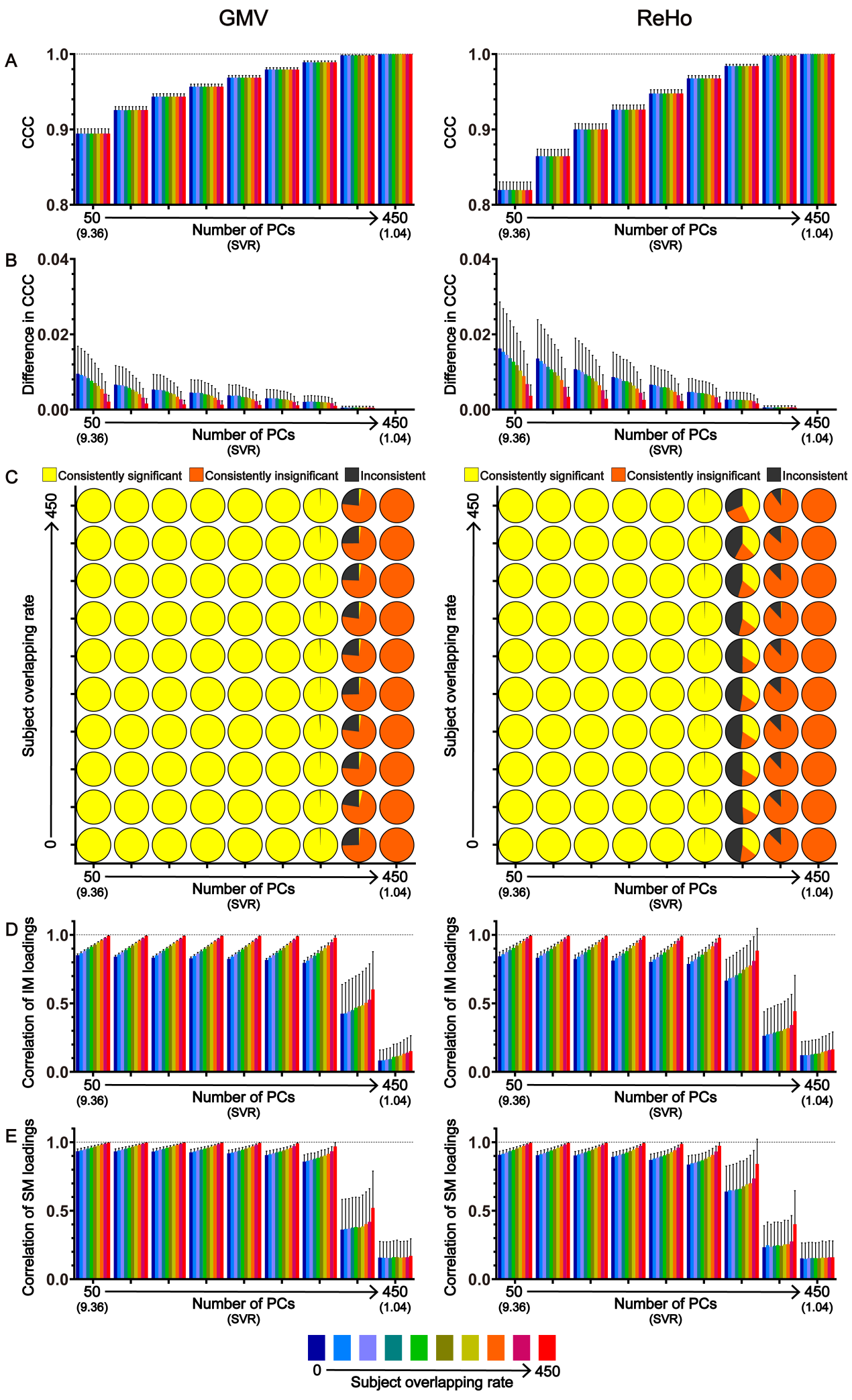
**

**Figure S13.** The results of CCA stability assessments using the Tianjin data set in the “control procedure” with all 78 subject measures (i.e., the “strong correlation” scenario). Panel A shows the magnitudes of CCCs obtained from 2,000 CCAs for all combinations of subject overlapping rate and data dimensionality. Panel B shows the absolute differences in CCCs of 1,000 pairs of CCA. Panel C shows the consistency of the statistical significance of CCCs between two subgroups of 1,000 pairs of CCAs. Panels D and E show the correlation coefficients of the loading vectors between two subgroups of 1,000 pairs of CCAs corresponding to brain imaging measures and those corresponding to subject measures, respectively. The abscissa of all subgraphs represents the dimensionality of imaging measures (i.e., the number of kept PCs, ranging from 50 to 450 with a step of 50) and the corresponding SVR (i.e., the ratio of the sample size to the dimensionality of the imaging measures, ranging from 9.36 to 1.04). The subject overlapping rates between two subgroups of each pair (ranging from 0 to 450 with a step of 50) are color coded. In all bar plots, the height of the bars indicates the mean and the error bars indicate the standard deviation.


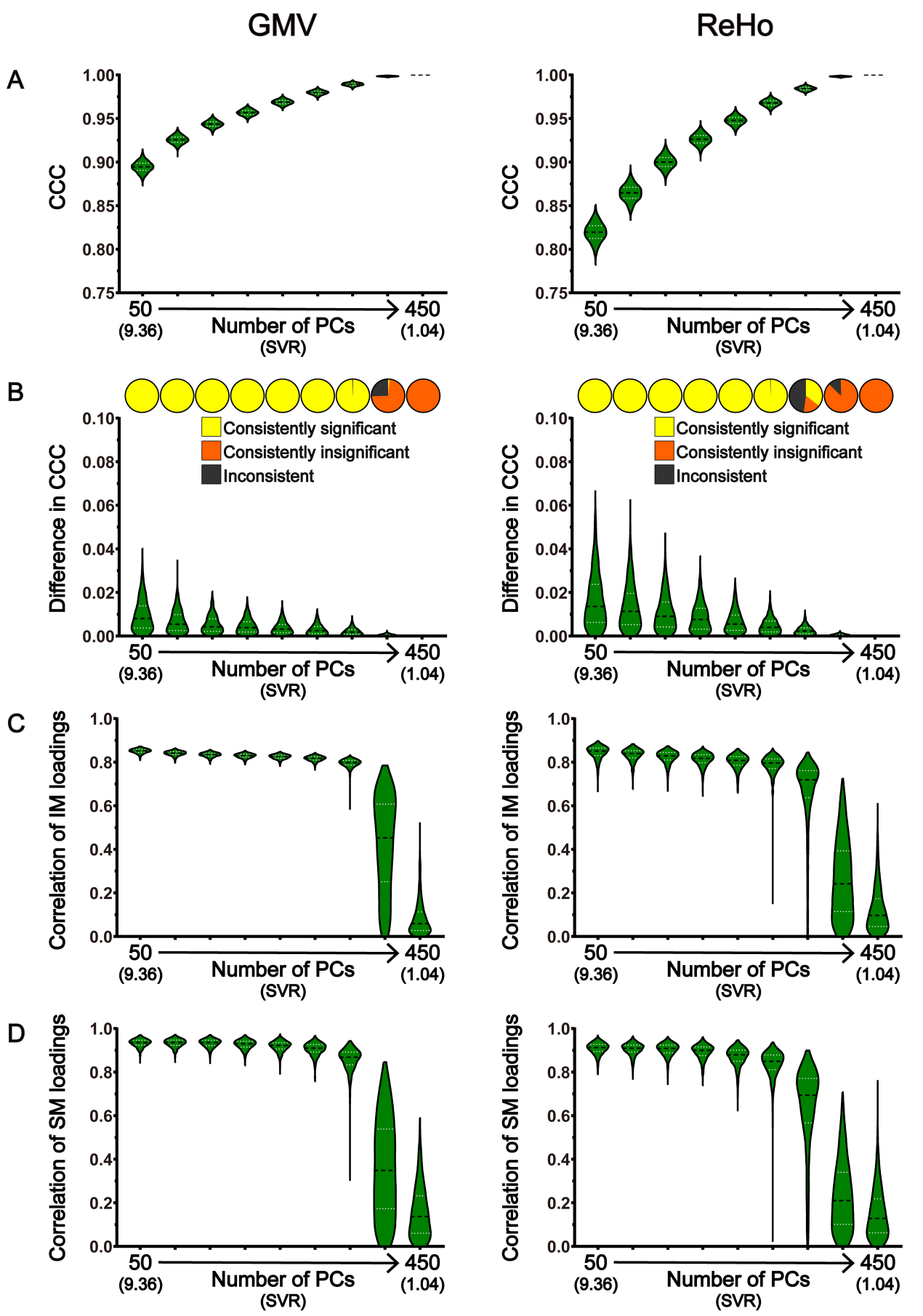


**Figure S14.** The results of CCA stability assessments using the Tianjin data set in the “control procedure” with all 78 subject measures (i.e., the “strong correlation” scenario) when there are no overlapping subjects between the two subgroups of 1,000 pairs of CCAs. Panel A shows the magnitudes of CCCs obtained from 2,000 CCAs for all data dimensionalities. Panel B shows the absolute differences in CCCs of 1,000 pairs of CCA (lower part) and the consistency of the statistical significance of CCCs between two subgroups of 1,000 pairs of CCAs (upper part). Panels C and D show the correlation coefficients of the loading vectors between two subgroups of 1,000 pairs of CCAs corresponding to brain imaging measures and those corresponding to subject measures, respectively. The abscissa of all subgraphs represents the dimensionality of imaging measures (i.e., the number of kept PCs, ranging from 50 to 450 with a step of 50) and the corresponding SVR (i.e., the ratio of the sample size to the dimensionality of the imaging measures, ranging from 9.36 to 1.04). In all smoothed violin plots, the sample median (black dotted line) and quartiles (white dotted line) are superimposed.


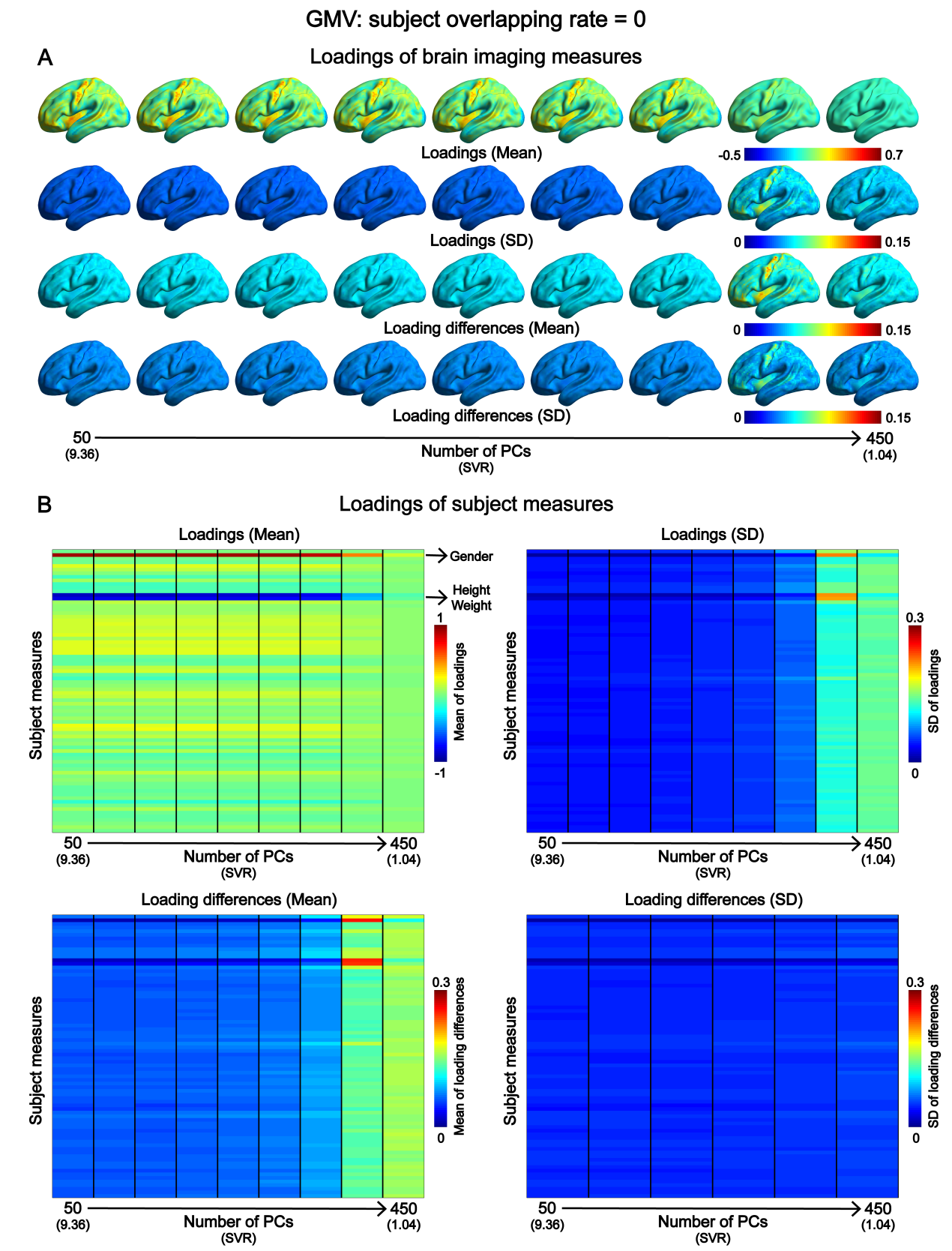


**Figure S15.** The results of loadings of brain imaging measures (panel A) and subject measures (panel B) of CCA between GMV and subject measures when there are no overlapping subjects between the two subgroups of 1,000 pairs of CCAs using Tianjin data set in the “control procedure” with all 78 subject measures (i.e., the “strong correlation” scenario). The mean and the standard deviation (SD) of the loadings across 2,000 CCAs are shown in the upper two rows in Panel A for brain imaging measures and in the upper part of Panel B for subject measures. The mean and the SD of the absolute differences in loadings between two paired subgroups of 1,000 pairs are shown in the lower two rows in Panel A for brain imaging measures and in the lower part of Panel B for subject measures. In panel A, loadings are arranged in brain space and for all dimensionalities of imaging measures ranging from 50 to 450 with a step of 50 (increasing from left to right) and the corresponding SVRs ranging from 9.36 to 1.04 (decreasing from left to right). In panel B, loadings are arranged in the form of a matrix with rows indicating variables and columns indicating dimensionalities and the corresponding SVRs.


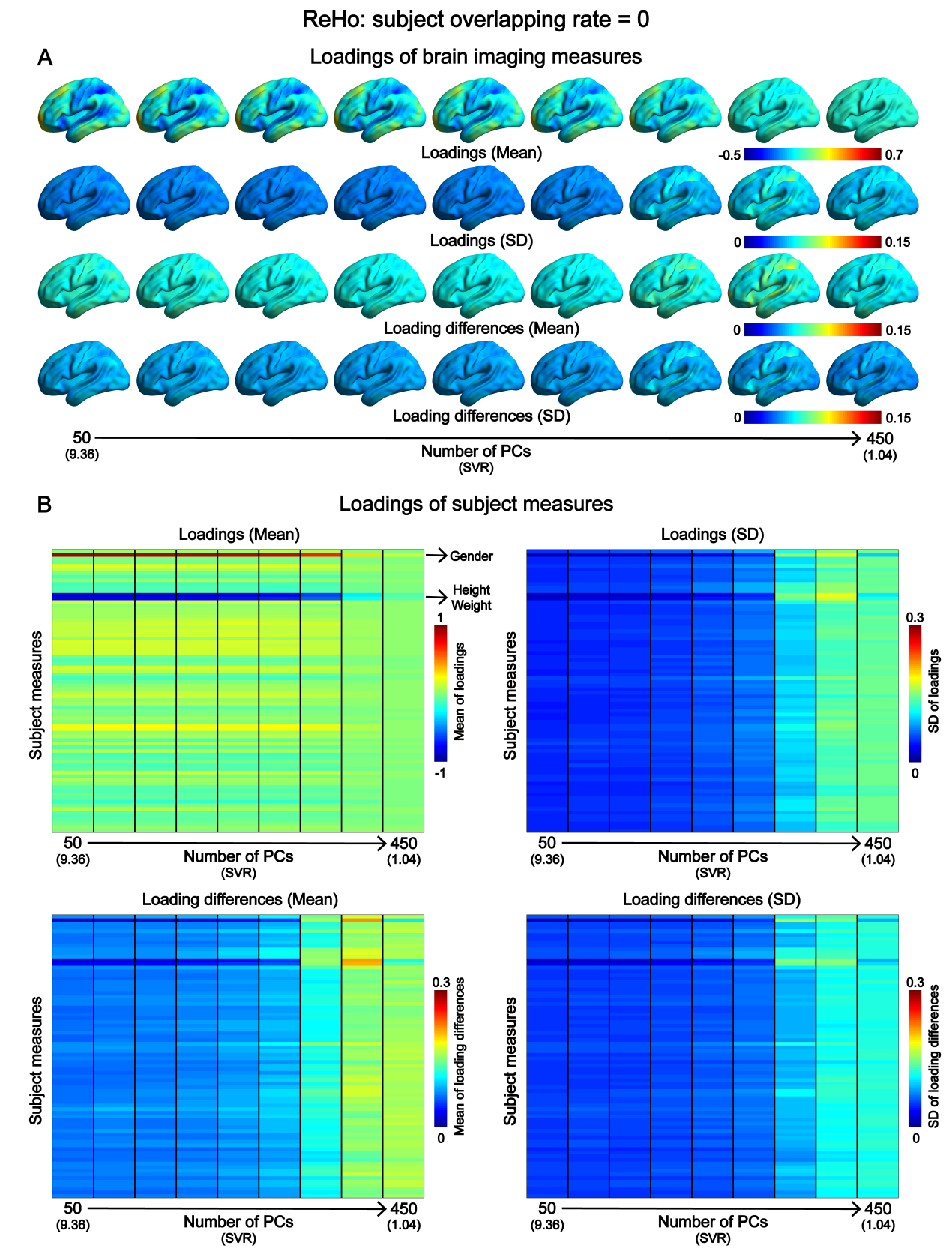


**Figure S16.** The results of loadings of brain imaging measures (panel A) and subject measures (panel B) of CCA between ReHo and subject measures when there are no overlapping subjects between the two subgroups of 1,000 pairs of CCAs using Tianjin data set in the “control procedure” with all 78 subject measures (i.e., the “strong correlation” scenario). The mean and the standard deviation (SD) of the loadings across 2,000 CCAs are shown in the upper two rows in Panel A for brain imaging measures and in the upper part of Panel B for subject measures. The mean and the SD of the absolute differences in loadings between two paired subgroups of 1,000 pairs are shown in the lower two rows in Panel A for brain imaging measures and in the lower part of Panel B for subject measures. In panel A, loadings are arranged in brain space and for all dimensionalities of imaging measures ranging from 50 to 450 with a step of 50 (increasing from left to right) and the corresponding SVRs ranging from 9.36 to 1.04 (decreasing from left to right). In panel B, loadings are arranged in the form of a matrix with rows indicating variables and columns indicating dimensionalities and the corresponding SVRs.


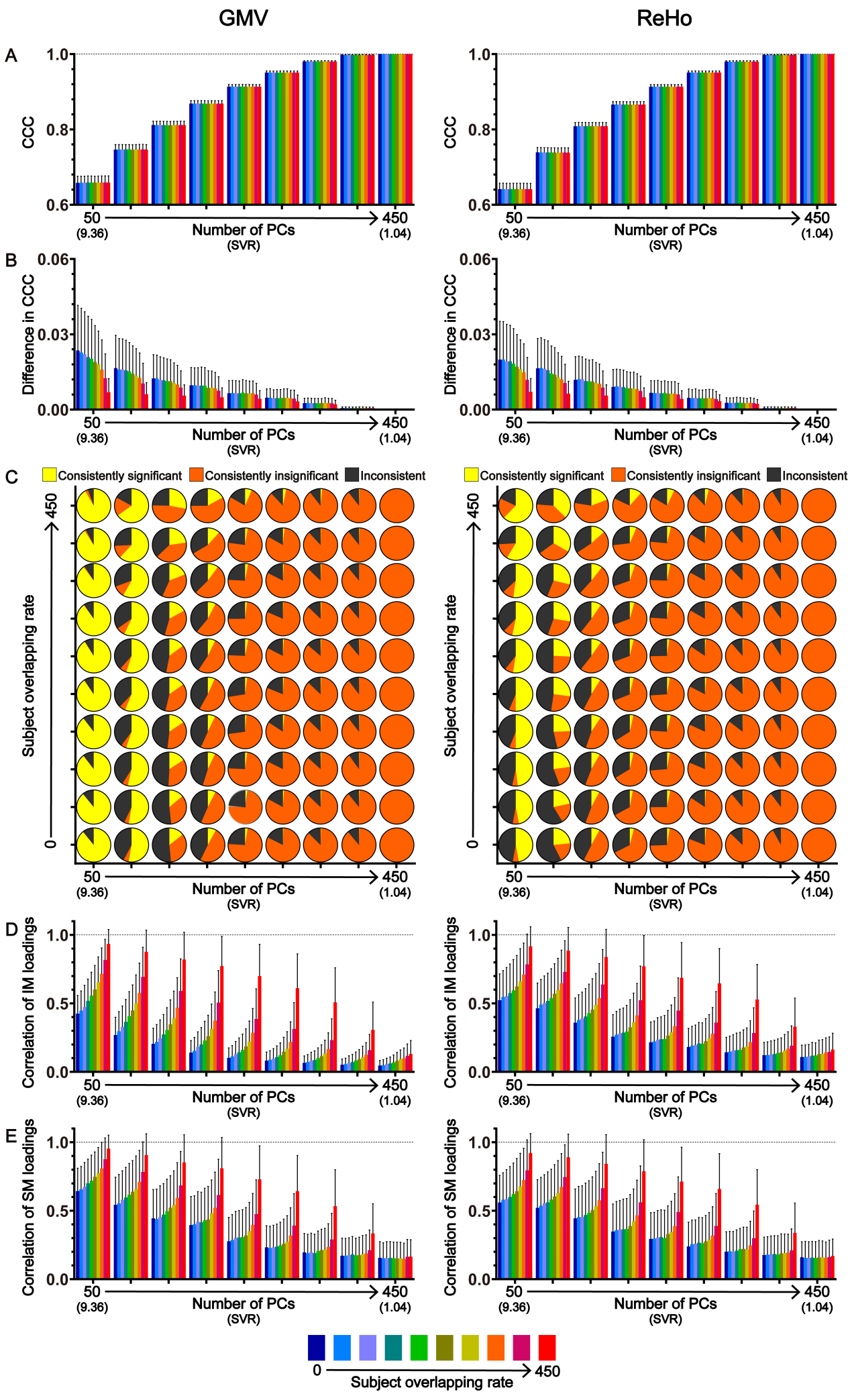


**Figure S17.** The results of CCA stability assessments using the Tianjin data set in the “control procedure” with 75 subject measures (i.e., the “moderate correlation” scenario). Panel A shows the magnitudes of CCCs obtained from 2,000 CCAs for all combinations of subject overlapping rate and data dimensionality. Panel B shows the absolute differences in CCCs of 1,000 pairs of CCA. Panel C shows the consistency of the statistical significance of CCCs between two subgroups of 1,000 pairs of CCAs. Panels D and E show the correlation coefficients of the loading vectors between two subgroups of 1,000 pairs of CCAs corresponding to brain imaging measures and those corresponding to subject measures, respectively. The abscissa of all subgraphs represents the dimensionality of imaging measures (i.e., the number of kept PCs, ranging from 50 to 450 with a step of 50) and the corresponding SVR (i.e., the ratio of the sample size to the dimensionality of the imaging measures, ranging from 9.36 to 1.04). The subject overlapping rates between two subgroups of each pair (ranging from 0 to 450 with a step of 50) are color coded. In all bar plots, the height of the bars indicates the mean and the error bars indicate the standard deviation.


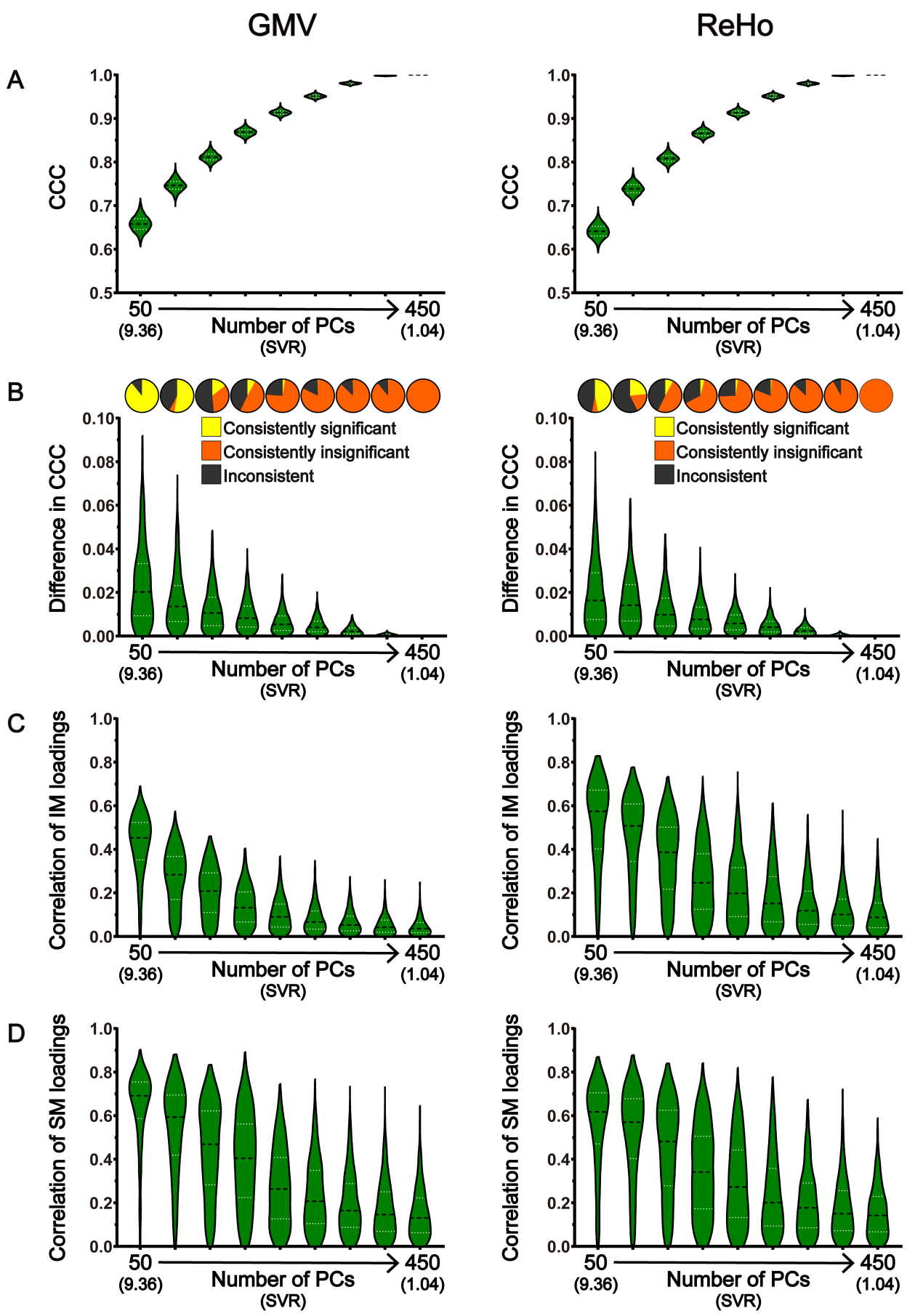


**Figure S18.** The results of CCA stability assessments using the Tianjin data set in the “control procedure” with all 75 subject measures (i.e., the “moderate correlation” scenario) when there are no overlapping subjects between the two subgroups of 1,000 pairs of CCAs. Panel A shows the magnitudes of CCCs obtained from 2,000 CCAs for all data dimensionalities. Panel B shows the absolute differences in CCCs of 1,000 pairs of CCA (lower part) and the consistency of the statistical significance of CCCs between two subgroups of 1,000 pairs of CCAs (upper part). Panels C and D show the correlation coefficients of the loading vectors between two subgroups of 1,000 pairs of CCAs corresponding to brain imaging measures and those corresponding to subject measures, respectively. The abscissa of all subgraphs represents the dimensionality of imaging measures (i.e., the number of kept PCs, ranging from 50 to 450 with a step of 50) and the corresponding SVR (i.e., the ratio of the sample size to the dimensionality of the imaging measures, ranging from 9.36 to 1.04). In all smoothed violin plots, the sample median (black dotted line) and quartiles (white dotted line) are superimposed.


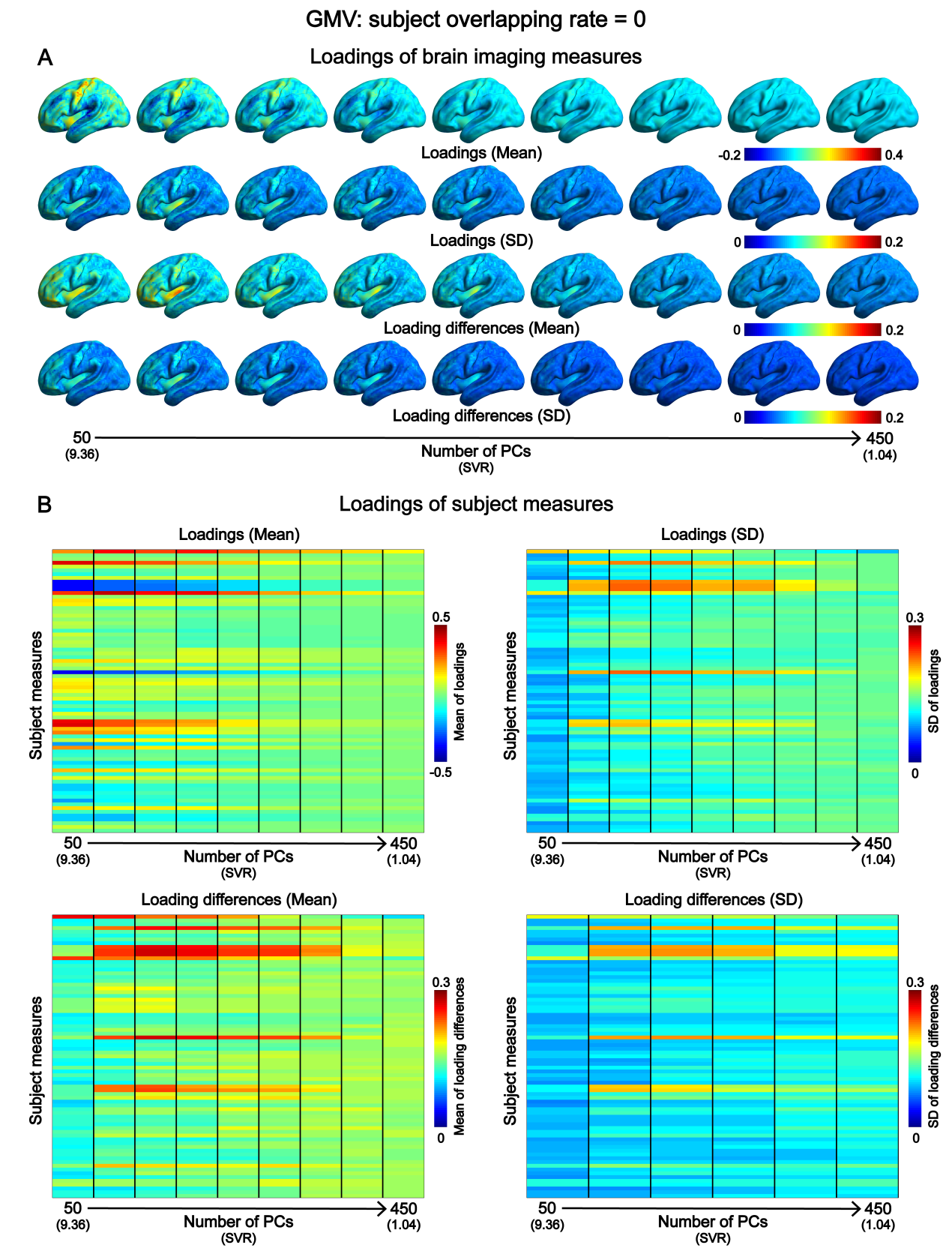


**Figure S19.** The results of loadings of brain imaging measures (panel A) and subject measures (panel B) of CCA between GMV and subject measures when there are no overlapping subjects between the two subgroups of 1,000 pairs of CCAs using Tianjin data set in the “control procedure” with 75 subject measures (i.e., the “moderate correlation” scenario). The mean and the standard deviation (SD) of the loadings across 2,000 CCAs are shown in the upper two rows in Panel A for brain imaging measures and in the upper part of Panel B for subject measures. The mean and the SD of the absolute differences in loadings between two paired subgroups of 1,000 pairs are shown in the lower two rows in Panel A for brain imaging measures and in the lower part of Panel B for subject measures. In panel A, loadings are arranged in brain space and for all dimensionalities of imaging measures ranging from 50 to 450 with a step of 50 (increasing from left to right) and the corresponding SVRs ranging from 9.36 to 1.04 (decreasing from left to right). In panel B, loadings are arranged in the form of a matrix with rows indicating variables and columns indicating dimensionalities and the corresponding SVRs.


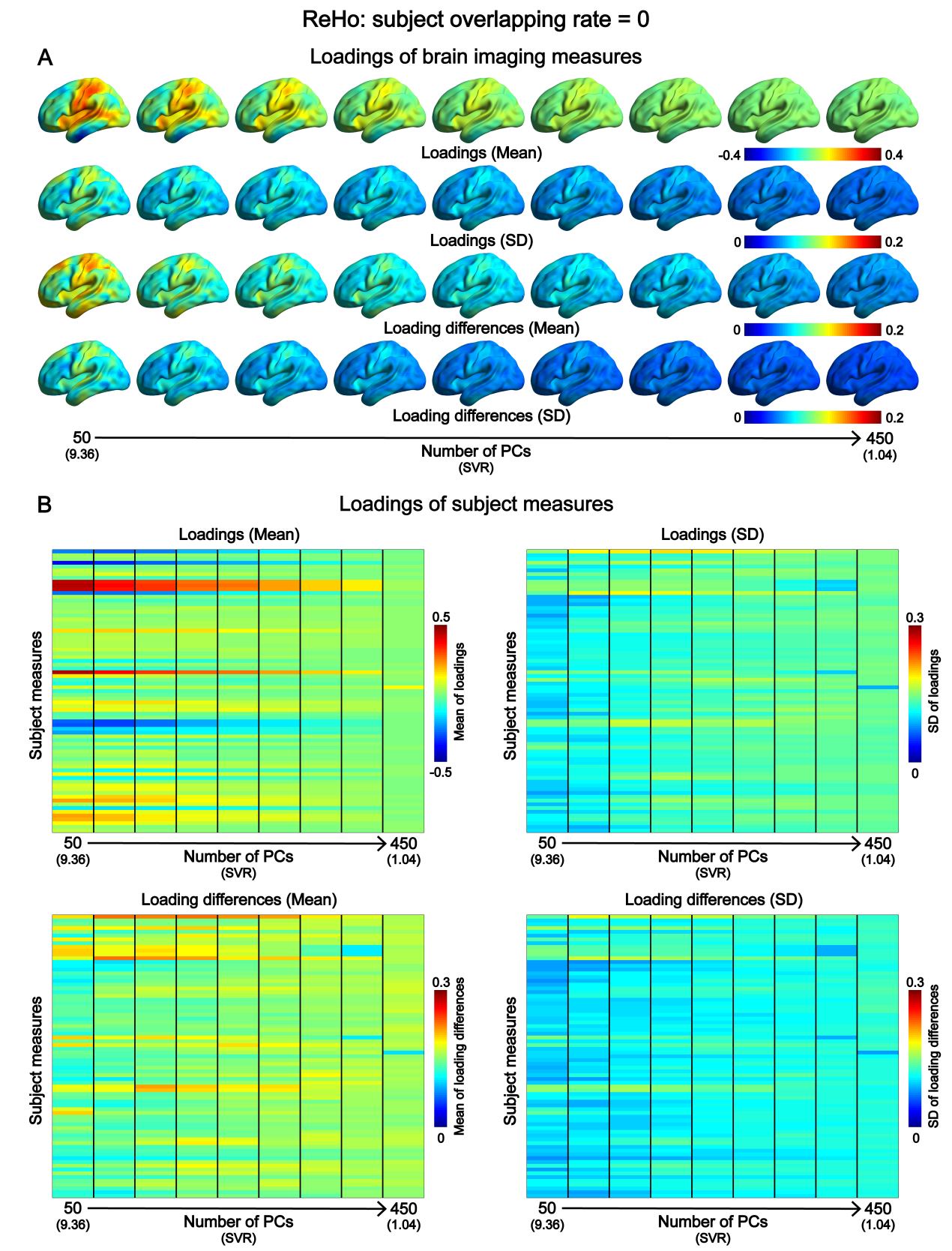


**Figure S20.** The results of loadings of brain imaging measures (panel A) and subject measures (panel B) of CCA between ReHo and subject measures when there are no overlapping subjects between the two subgroups of 1,000 pairs of CCAs using Tianjin data set in the “control procedure” with 75 subject measures (i.e., the “moderate correlation” scenario). The mean and the standard deviation (SD) of the loadings across 2,000 CCAs are shown in the upper two rows in Panel A for brain imaging measures and in the upper part of Panel B for subject measures. The mean and the SD of the absolute differences in loadings between two paired subgroups of 1,000 pairs are shown in the lower two rows in Panel A for brain imaging measures and in the lower part of Panel B for subject measures. In panel A, loadings are arranged in brain space and for all dimensionalities of imaging measures ranging from 50 to 450 with a step of 50 (increasing from left to right) and the corresponding SVRs ranging from 9.36 to 1.04 (decreasing from left to right). In panel B, loadings are arranged in the form of a matrix with rows indicating variables and columns indicating dimensionalities and the corresponding SVRs.


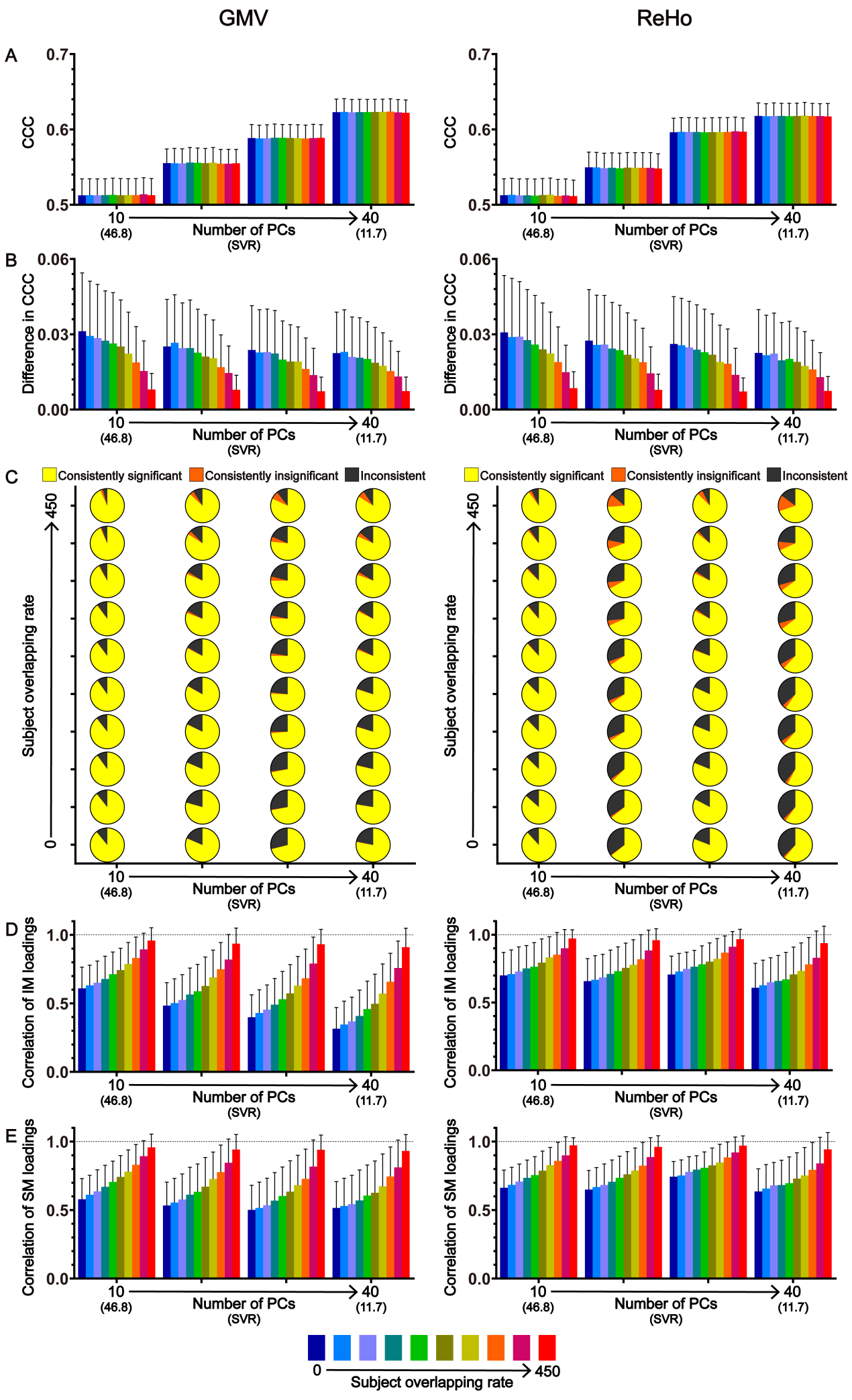


**Figure S21.** The results of CCA stability assessments using the Tianjin data set in the “control procedure” with 75 subject measures (i.e., the “moderate correlation” scenario). Panel A shows the magnitudes of CCCs obtained from 2,000 CCAs for all combinations of subject overlapping rate and data dimensionality. Panel B shows the absolute differences in CCCs of 1,000 pairs of CCA. Panel C shows the consistency of the statistical significance of CCCs between two subgroups of 1,000 pairs of CCAs. Panels D and E show the correlation coefficients of the loading vectors between two subgroups of 1,000 pairs of CCAs corresponding to brain imaging measures and those corresponding to subject measures, respectively. The abscissa of all subgraphs represents the dimensionality of imaging measures (i.e., the number of kept PCs, ranging from 10 to 40 with a step of 10) and the corresponding SVR (i.e., the ratio of the sample size to the dimensionality of the imaging measures, ranging from 46.8 to 11.7). The subject overlapping rates between two subgroups of each pair (ranging from 0 to 450 with a step of 50) are color coded. In all bar plots, the height of the bars indicates the mean and the error bars indicate the standard deviation.


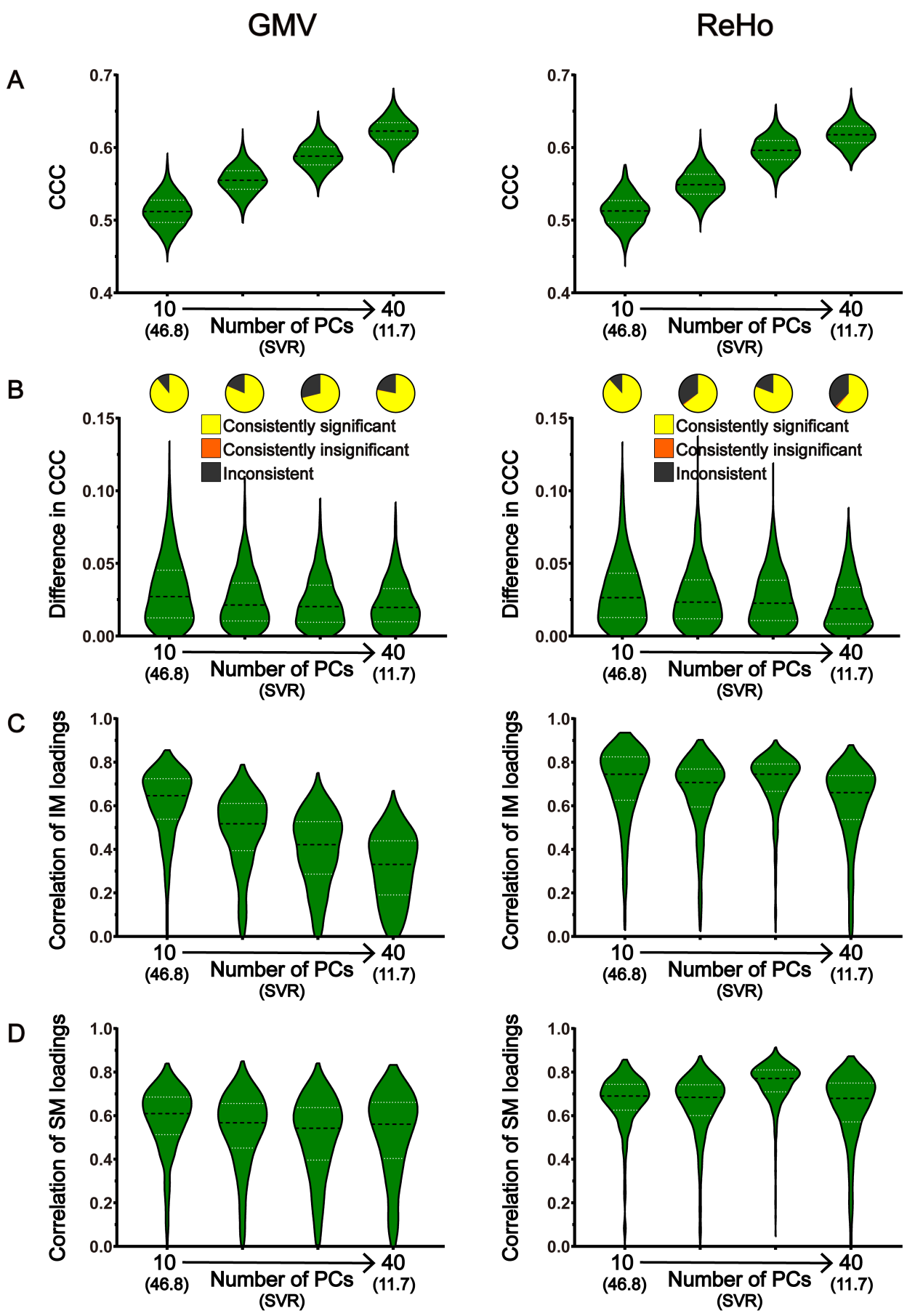


**Figure S22.** The results of CCA stability assessments using the Tianjin data set in the “control procedure” with 75 subject measures (i.e., the “moderate correlation” scenario) when there are no overlapping subjects between the two subgroups of 1,000 pairs of CCAs. Panel A shows the magnitudes of CCCs obtained from 2,000 CCAs for all data dimensionalities. Panel B shows the absolute differences in CCCs of 1,000 pairs of CCA (lower part) and the consistency of the statistical significance of CCCs between two subgroups of 1,000 pairs of CCAs (upper part). Panels C and D show the correlation coefficients of the loading vectors between two subgroups of 1,000 pairs of CCAs corresponding to brain imaging measures and those corresponding to subject measures, respectively. The abscissa of all subgraphs represents the dimensionality of imaging measures (i.e., the number of kept PCs, ranging from 10 to 40 with a step of 10) and the corresponding SVR (i.e., the ratio of the sample size to the dimensionality of the imaging measures, ranging from 46.8 to 11.7). In all smoothed violin plots, the sample median (black dotted line) and quartiles (white dotted line) are superimposed.


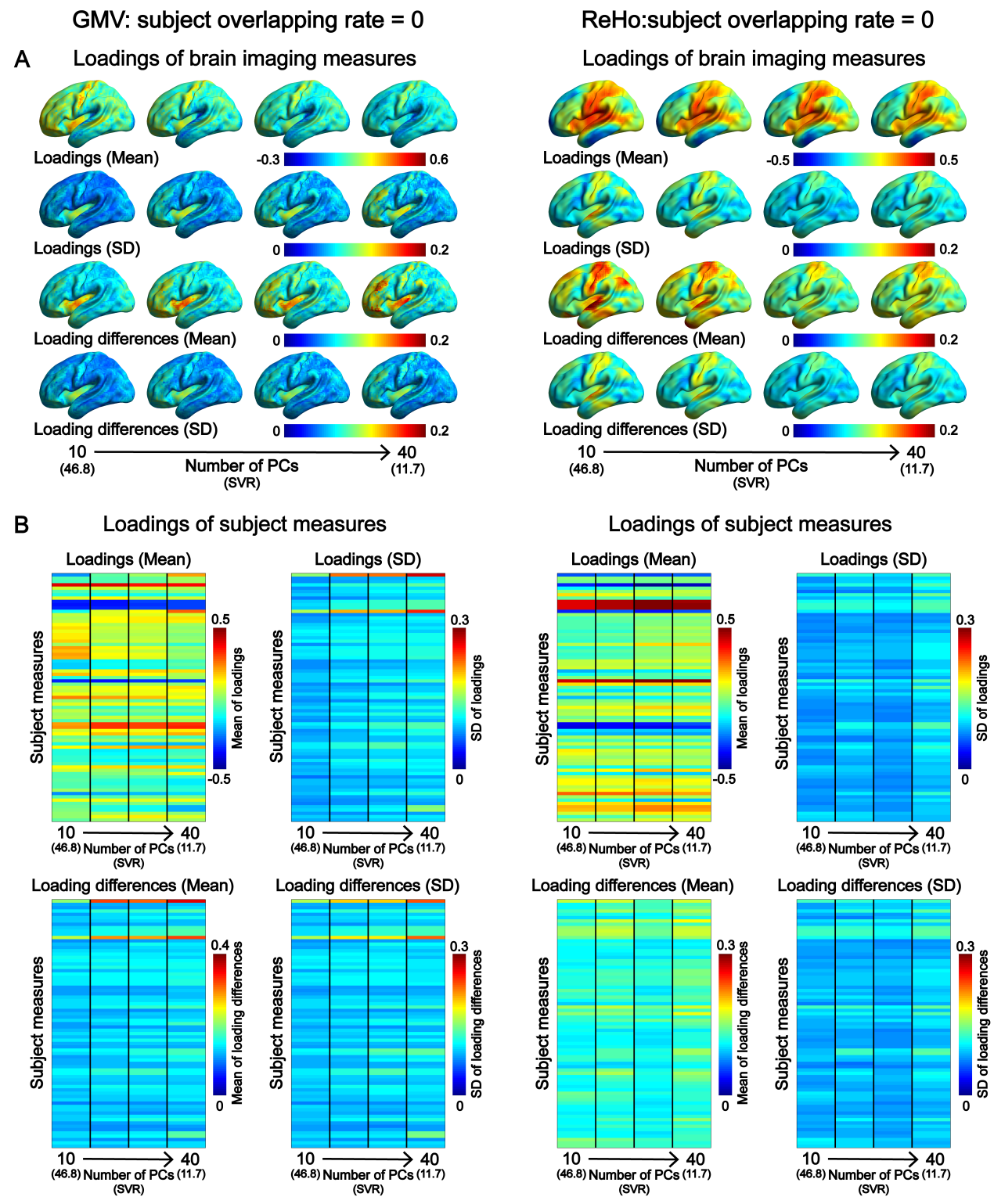


**Figure S23.** The results of loadings of brain imaging measures and subject measures of CCA between GMV and subject measures (left panel) and between ReHo and subject measures (right panel) for extremely low dimensionalities (i.e., 10, 20, 30 and 40) when there are no overlapping subjects between the two subgroups of 1,000 pairs of CCAs using Tianjin data set in the “control procedure” with 75 subject measures (i.e., the “moderate correlation” scenario). The mean and the standard deviation (SD) of the loadings across 2,000 CCAs are shown in the upper two rows in Panel A for brain imaging measures and in the upper part of Panel B for subject measures. The mean and the SD of the absolute differences in loadings between two paired subgroups of 1,000 pairs are shown in the lower two rows in Panel A for brain imaging measures and in the lower part of Panel B for subject measures. In panel A, loadings are arranged in brain space and for extremely low dimensionalities of imaging measures ranging from 10 to 40 with a step of 10 (increasing from left to right) and the corresponding SVRs ranging from 46.8 to 11.7 (decreasing from left to right). In panel B, loadings are arranged in the form of a matrix with rows indicating variables and columns indicating dimensionalities and the corresponding SVRs.


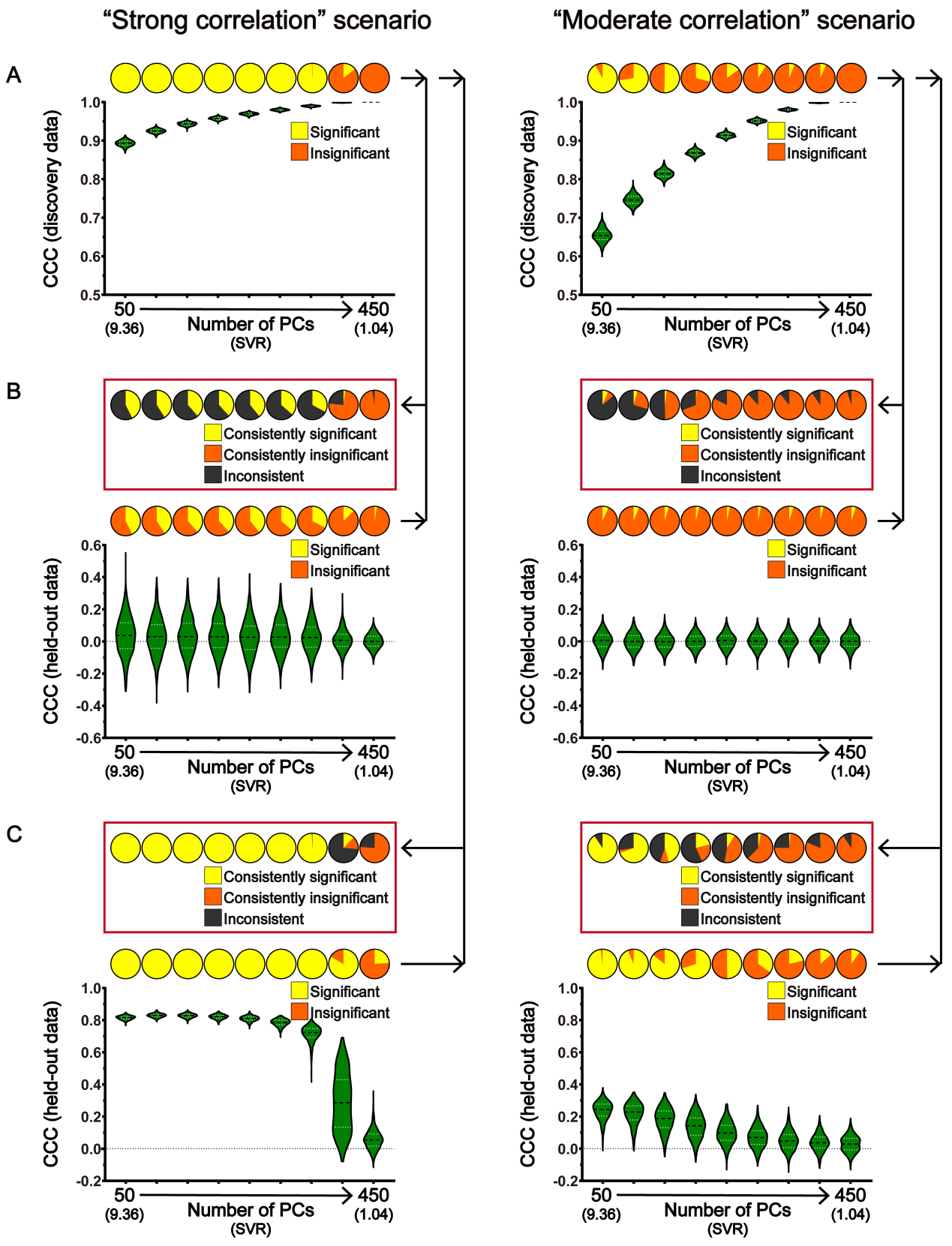


**Figure S24.** Panel A shows the magnitudes of CCCs (the violin plots) and their statistical significance (the pie charts) across 1,000 discovery sets for all data dimensionalities. Panel B shows the results of the held-out data when PCA was applied separately to the discovery data and the held-out data: the magnitudes of CCCs (the violin plots), their statistical significance (the pie charts above the violin plots) and the consistency of statistical significance between the discovery data and the held-out data (the pie charts inside the red rectangle at the top) across 1,000 repetitions for all data dimensionalities. Panel C shows the results of the held-out data when PCA was only applied to the discovery data and the same transformation matrix was applied to the held-out data to remove possible inconsistency of PCs between the two datasets: the magnitudes of CCCs (the violin plots), their statistical significance (the pie charts above the violin plots) and the consistency of statistical significance between the discovery data and the held-out data (the pie charts inside the red rectangle at the top) across 1,000 repetitions for all data dimensionalities. The results in the “strong correlation” scenario and the “moderate correlation” scenario are shown in the left column and the right column, respectively. In all violin plots, the black dotted line indicates the median and the white dotted lines indicate the quartiles. This figure shows that, without removing the effects of PC inconsistency between the discovery data and the held-out data, the resultant CCCs and their statistical significance were highly inconsistent between the discovery data and the held-out data for both “strong correlation” scenario and “moderate correlation” scenario (A&B): taking the results obtained when the imaging measures dimensionality = 50 as an example, for the “strong correlation” scenario, the CCCs were 0.8932±0.0075 (mean±SD) in the discovery data but decreased to 0.0353±0.1185 in the held-out data; for the “moderate correlation” scenario, the CCCs were 0.6545±0.0172 in the discovery data but decreased to 0.0039±0.0529 in the held-out data. In contrast, after removing the effects of PC inconsistency between the discovery data and the held-out data, the CCA results were generally stable in the “strong correlation” scenario when the imaging measures dimensionality ≤350 (i.e., similar CCCs and statistical significance between the discovery data and the held-out data), but became instable in the “moderate correlation” scenario (much lower CCCs in the held-out data compared with the discovery data), confirming again that the CCA results were instable when the SVR is not sufficiently high or in the “moderate correlation” scenario (A&C).
